# Bax deficiency extends the survival of Ku70 knockout mice that develop lung and heart diseases

**DOI:** 10.1038/cddis.2015.11

**Published:** 2015-03-26

**Authors:** J Ngo, M Matsuyama, C Kim, I Poventud-Fuentes, A Bates, S L Siedlak, H-g Lee, Y Q Doughman, M Watanabe, A Liner, B Hoit, N Voelkel, S Gerson, P Hasty, S Matsuyama

**Affiliations:** 1 Department of Medicine, School of Medicine, Case Western Reserve University, Cleveland, OH, USA; 2 Department of Genetics and Genome Sciences, School of Medicine, Case Western Reserve University, Cleveland, OH, USA; 3 Department of Pathology, School of Medicine, Case Western Reserve University, Cleveland, OH, USA; 4 Department of Pediatrics, School of Medicine, Case Western Reserve University, Cleveland, OH, USA; 5 Pulmonary and Critical Care Medicine Division and Victoria Johnson Center for Pulmonary Obstructive Research, Virginia Commonwealth University, Richmond, VA, USA; 6 Department of Case Comprehensive Cancer Center, School of Medicine, Case Western Reserve University, Cleveland, OH, USA; 7 Department of Molecular Medicine and Institute of Biotechnology, University of Texas Health Science Center, San Antonio, TX, USA

## Abstract

Ku70 (Lupus Ku autoantigen p70) is essential in nonhomologous end joining DNA double-strand break repair, and *ku70*^*−/−*^ mice age prematurely because of increased genomic instability and DNA damage responses. Previously, we found that Ku70 also inhibits Bax, a key mediator of apoptosis. We hypothesized that Bax-mediated apoptosis would be enhanced in the absence of Ku70 and contribute to premature death observed in *ku70*^*−/−*^ mice. Here, we show that *ku70*^*−/−*^ *bax*^*+/−*^ and *ku70*^*−/−*^ *bax*^*−/−*^ mice have better survival, especially in females, than *ku70*^*−/−*^ mice, even though Bax deficiency did not decrease the incidence of lymphoma observed in a Ku70-null background. Moreover, we found that *ku70*^*−/−*^ mice develop lung diseases, like emphysema and pulmonary arterial (PA) occlusion, by 3 months of age. These lung abnormalities can trigger secondary health problems such as heart failure that may account for the poor survival of *ku70*^*−/−*^ mice. Importantly, Bax deficiency appeared to delay the development of emphysema. This study suggests that enhanced Bax activity exacerbates the negative impact of Ku70 deletion. Furthermore, the underlying mechanisms of emphysema and pulmonary hypertension due to PA occlusion are not well understood, and therefore *ku70*^*−/−*^ and Bax-deficient *ku70*^*−/−*^ mice may be useful models to study these diseases.

Ku70 (Lupus Ku autoantigen p70) is a subunit of the Ku protein complex that plays an essential role in the nonhomologous end joining (NHEJ) pathway for DNA double-strand break (DSB) repair (reviewed in Downs and Jackson^1^). Ku70-null mice have an increased sensitivity to radiation and are defective in lymphocyte differentiation because of a lack of NHEJ-dependent DSB repair.^2, 3, 4, 5^ Recent studies have shown that Ku70-null mice exhibit characteristics of premature aging,^6^ some of which may arise as a result of the cellular responses to increased DNA damage caused by defective DNA repair. Furthermore, increased neuronal cell death observed during the development of *ku70*^*−/−*^ mice^7^ suggests that Ku70-dependent DNA repair may be essential for cell survival during normal brain development. There is increasing evidence that Ku70 has multiple biological activities independent of its role in the nucleus as a subunit of the Ku protein complex.^1^

Ku70 is known to have anti-apoptotic activity by suppressing the intrinsic cell death pathway^8^ mediated by the pro-apoptotic Bcl-2 family of proteins, such as Bax (Bcl-2-associated protein X).^9, 10^ Ku70-deficient cells have increased sensitivities to apoptotic stresses that are not limited to DNA-damaging stresses,^8, 11, 12^ consistent with its anti-apoptotic activity. Our previous studies suggest that Ku70 can interact with Bax to inhibit the conformational change required for Bax-induced apoptosis.^8^ To date, several studies have shown that regulating the interaction between Ku70 and Bax in the cytosol can influence the cellular sensitivity to various types of stresses.^12, 13, 14, 15, 16, 17, 18, 19, 20, 21, 22, 23, 24, 25, 26, 27, 28^ Recent studies have also shown that Ku70 can suppress apoptosis by maintaining anti-apoptotic Mcl-1 protein levels through deubiquitinization,^29^ suppressing Apaf-1 (apoptosis protease activation factor-1) transcription^30^ and inhibiting p18-Cyclin E-induced cell death produced by the caspase-dependent cleavage of cyclin E.^17^

To determine the physiological significance of Ku70-dependent inhibition of Bax-mediated apoptosis, *ku70*^*−/−*^ *bax*^*+/−*^ and *ku70*^*−/−*^ *bax*^*−/−*^ mice were generated and their phenotypes were compared with *ku70*^*−/−*^ *bax*^*+/+*^ mice. In the absence of Ku70, we hypothesized that Bax-mediated apoptosis would be enhanced. We found that Bax deficiency decreased the mortality of *ku70*^*−/−*^ mice and improved the median and maximum age of survival. This study also provides evidence that apoptosis has a significant role in age-associated degenerative diseases in defective DNA repair mutant mice.

## Results

### Bax deficiency improves the survival and lifespan in Ku70-null mice

**Figure 1:**
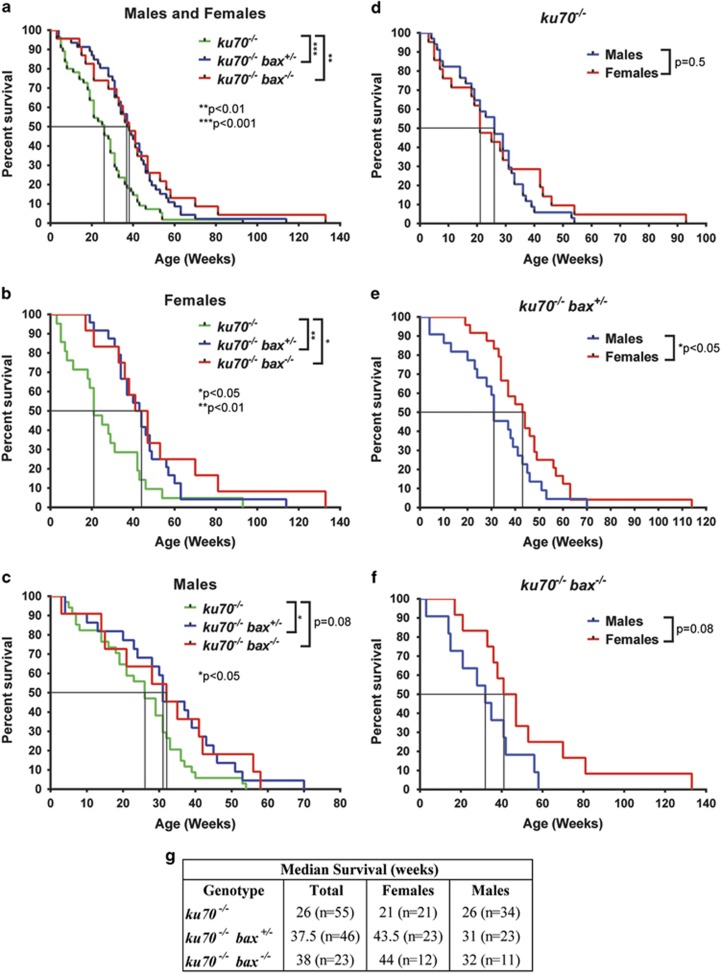
Bax deficiency improved the survival and extended the lifespan of Ku70-null mice. Kaplan–Meier survival curves are shown for (**a**) all mice analyzed, (**b**) females, and (**c**) males. Sex-specific survival are shown for (**d**) *ku70*^*−/−*^, (**e**) *ku70*^*−/−*^ *bax*^*+/−*^, and (**f**) *ku70*^*−/−*^ *bax*^*−/−*^. (**g**) The table summarizes the median survival of all mice analyzed

The *ku70*^*−/−*^ mice in this study exhibited shortened lifespans as previously reported,^6^ with a median survival of 26 weeks (*n*=55). The *ku70*^*+/+*^ (wild type) mice in the same cohort had a survival rate of 99.4% (*n*=355) at 26 weeks of age (data not shown), and it has been reported that the median lifespan of wild-type mice (C57BL/6J) is ∼110 weeks.^31^ In comparison with *ku70*^*−/−*^ mice, the median survival of *ku70*^*−/−*^ *bax*^*+/−*^ and *ku70*^*−/−*^ *bax*^*−/−*^ mice was significantly longer at 37.5 (*n*=46; *P*<0.001) and 38 (*n*=23; *P*<0.01) weeks, respectively (Figures 1a and g). The maximum lifespans of *ku70*^*−/−*^ *bax*^*+/−*^ (803 days, female) and *ku70*^*−/−*^ *bax*^*−/−*^ (951 days, female) mice were also greater than that of *ku70*^*−/−*^ mice (656 days, female) (Figures 1a and b). These results suggest that Bax deficiency was able to decrease the mortality of *ku70*^*−/−*^ mice that have been reported to develop premature aging phenotypes.^2, 3, 4, 5, 6^ The complete deletion of Bax may have a greater positive impact on the overall survival and lifespan of Ku70-null mice based on the lower observed mortality of *ku70*^*−/−*^ *bax*^*−/−*^ mice compared with *ku70*^*−/−*^ *bax*^*+/−*^ mice after 30 weeks of age. The extended survival because of Bax deficiency was also much more apparent in females than males (Figures 1b and c). Although there were no sex-dependent differences in the lifespan of *ku70*^*−/−*^ mice (Figure 1d), these differences became obvious in *ku70*^*−/−*^ *bax*^*+/−*^ and *ku70*^*−/−*^ *bax*^*−/−*^ mice (Figures 1e–g).

### Bax deficiency in Ku70-null mice leads to a slight increase in body weight and brain size

**Figure 2:**
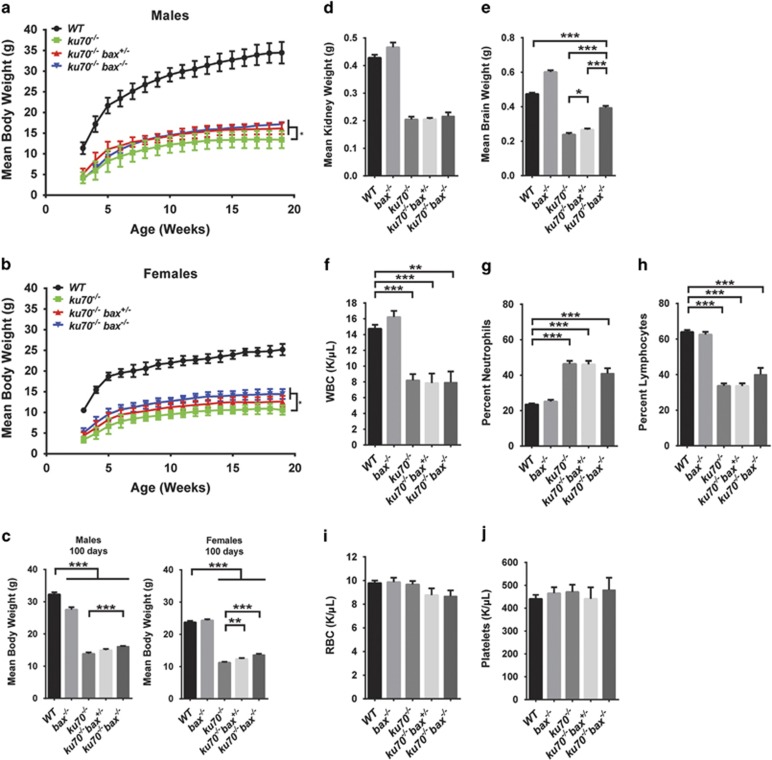
Growth curves, organ weights, and hematocrit analyses. (**a** and **b**) The growth curves shown are based on the average body weights measured in (**a**) male *WT* (*n*=5), *ku70*^*−/−*^ (*n*=9), *ku70*^*−/−*^ *bax*^*+/−*^ (*n*=8), and *ku70*^*−/−*^ *bax*^*−/−*^ (*n*=5) mice, and (**b**) female *WT* (*n*=6), *ku70*^*−/−*^ (*n*=6), *ku70*^*−/−*^ *bax*^*+/−*^ (*n*=11), and *ku70*^*−/−*^ *bax*^*−/−*^ (*n*=6) mice. (**c**) The mean body weights are shown for 100 days of age for *WT* (males *n*=11; females *n*=10), *bax*^*−/−*^ (males *n*=7; females *n*=3), *ku70*^*−/−*^ (males *n*=17; females *n*=13), *ku70*^*−/−*^ *bax*^*+/−*^ (males *n*=7; females *n*=17), and *ku70*^*−/−*^ *bax*^*−/−*^ (males *n*=7; females *n*=9) mice. The average weights are shown for (**d**) kidney in *WT* (*n*=35), *bax*^*−/−*^ (*n*=51), *ku70*^*−/−*^ (*n*=9), *ku70*^*−/−*^ *bax*^*+/−*^ (*n*=9), and *ku70*^*−/−*^ *bax*^*−/−*^ (*n*=5) mice and (**e**) brain in *WT* (*n*=18), *bax*^*−/−*^ (*n*=35), *ku70*^*−/−*^ (*n*=9), *ku70*^*−/−*^ *bax*^*+/−*^ (*n*=12), and *ku70*^*−/−*^ *bax*^*−/−*^ (*n*=5) mice. (**f–j**) Hematocrit quantification of (**f**) WBCs and the percent composition of (**g**) neutrophils and (**h**) lymphocytes, (**i**) RBCs, and (**j**) platelets; *WT* (*n*=97), *bax*^*−/−*^ (*n*=43), *ku70*^*−/−*^ (*n*=20), *ku70*^*−/−*^ *bax*^*+/−*^ (*n*=15), and *ku70*^*−/−*^ *bax*^*−/−*^ (*n*=6). *P<0.05, **P<0.01, ***P<0.0001

Similar to previous reports,^6^ *ku70*^*−/−*^ mice displayed growth retardation. However, the average body weight was slightly increased in *ku70*^*−/−*^ *bax*^*+/−*^ and *ku70*^*−/−*^ *bax*^*−/−*^ mice (Figures 2a–c). To determine whether Bax deficiency affected specific organs to increase the overall body weight, we measured the weights of the kidneys and brain (Figures 2d and e). We found that the average weights of the kidneys in *ku70*^*−/−*^ *bax*^*+/−*^ and *ku70*^*−/−*^ *bax*^*−/−*^ mice were not significantly increased compared with *ku70*^*−/−*^ mice (Figure 2d). However, the average brain weight was significantly increased in *ku70*^*−/−*^ *bax*^*−/−*^ (*P*<0.0001) mice, and to a lesser extent in *ku70*^*−/−*^ *bax*^*+/−*^ (*P*<0.05) mice (Figure 2e), even though *ku70*^*−/−*^ *bax*^*+/−*^ and *ku70*^*−/−*^ *bax*^*−/−*^ mice have similar extended survival. This suggests that changes in brain size may not necessarily alter lifespan. Bax-null mice are known to have increased brain weight (Figure 2e) because of the suppression of neuronal cell death during development ^32^, whereas *ku70*^*−/−*^ mice exhibit increased neuronal cell death.^7^ The effects of Bax deletion on the brain weight in Ku70-null mice in this study are consistent with these previous reports.

Previous studies have reported that *ku70*^*−/−*^ mice have decreased lymphocyte counts because of deficient lymphocyte differentiation caused by the absence of NHEJ-dependent T-cell and B-cell receptor maturation.^2, 3, 4, 5^ Our blood cell count measurements were consistent with these findings (Figures 2f–j). The number of neutrophils was increased in *ku70*^*−/−*^ mice (Figure 2g), most likely to compensate for the decrease in lymphocytes (Figure 2h). Bax deficiency did not significantly affect the overall number of white and red blood cells and platelets in Ku70-null mice (Figures 2f, i, and j).

### Bax deficiency in Ku70-null MEFs decreases apoptosis sensitivity but does not affect cellular senescence *in vitro*

**Figure 3:**
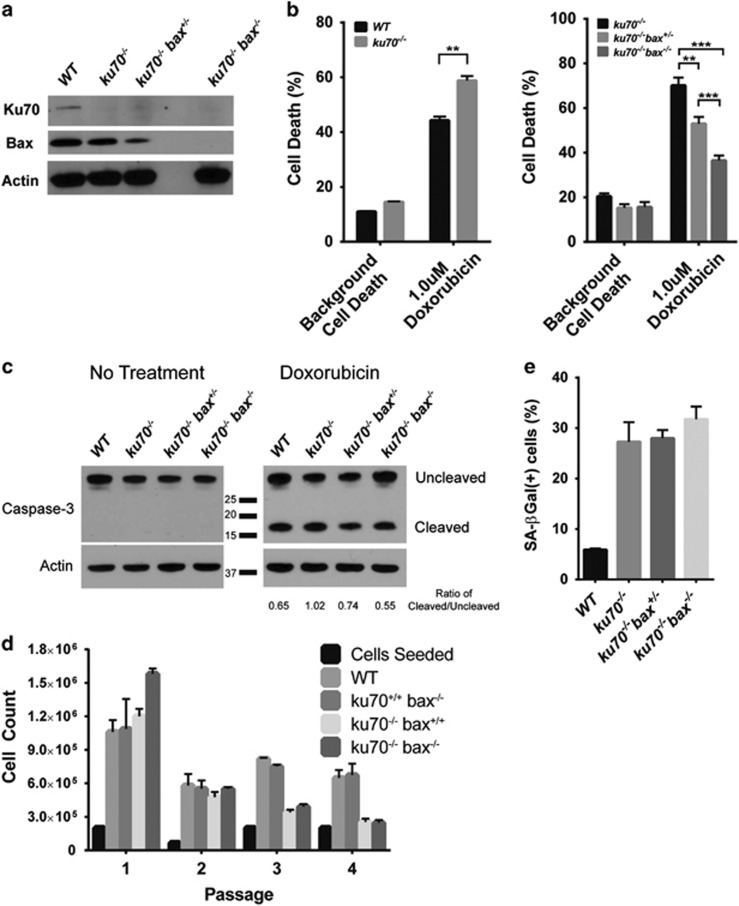
Bax deficiency reduced *ku70*^*−/−*^ MEF sensitivity to apoptosis but not cellular senescence. (**a**) Immunoblots of MEFs showing the protein expression levels of Ku70, Bax, and actin. (**b**) In comparison with *WT*, *ku70*^*−/−*^ MEFs showed increased apoptosis sensitivity to Doxorubicin, whereas Bax-deficient Ku70-null MEFs displayed decreased Doxorubicin-induced apoptosis (*n*=4). (**c**) Doxorubicin treatment led to Caspase-3 cleavage. Densimetric analyses are shown for independent experiments (*n*=2). Based on (**d**) cell counts after each passage (*n*=4) and the quantification of (**e**) senescence-associated *β*-galactosidase staining (*n*=4), Bax deficiency did not delay cellular senescence in *Ku70-*null MEFs. **P<0.01, ***P<0.0001

Ku70-null and Bax mutant mouse embryonic fibroblasts (MEFs) were used to determine whether cell death susceptibility could be attenuated by Bax deficiency (Figures 3a–c). As previously reported,^8, 12, 16, 33^ *ku70*^*−/−*^ MEFs show increased sensitivity to Doxorubicin, which induces DNA damage and activates Bax-mediated apoptosis. We confirmed that Bax deficiency attenuated cell death induction by Doxorubicin treatment in *ku70*^*−/−*^ *bax*^*+/−*^ and *ku70*^*−/−*^ *bax*^*−/−*^ MEFs (Figures 3b and c). We further examined whether Bax deficiency influences cellular senescence in *ku70*^*−/−*^ *bax*^*+/−*^ and *ku70*^*−/−*^ *bax*^*−/−*^ MEFs (Figures 3d and e). As previously reported,^34^ *ku70*^*−/−*^ MEFs undergo fewer passages and reach cellular senescence earlier than wild-type MEFs (Figure 3d). Bax deficiency did not show a significant effect on cellular senescence in *ku70*^*−/−*^ *bax*^*+/−*^ and *ku70*^*−/−*^ *bax*^*−/−*^ MEFs based on decreased cell counts after multiple passages (Figure 3d) and similar senescence associated *β*-galactosidase staining as *ku70*^*−/−*^ MEFs (Figure 3e).

### Cancer incidence and abnormalities in moribund or dead mice

Almost one-third (28.6%) of *ku70*^*−/−*^ mice developed thymic tumors or disseminated lymphoma with splenomegaly and hepatomegaly (Table 1 and Supplementary Figure S1). A slight difference in the development of lymphoma was observed between *ku70*^*−/−*^ males (27.3%, *n*=33) and females (33.3%, *n*=21) (Supplementary Table S1). Two other independent studies previously reported T-cell lymphomas in *ku70*^*−/−*^ C57BL/6J mice,^3, 4^ but not in the 129xC57BL/6 strain,^6^ suggesting that the development of T-cell lymphoma is strain dependent. In the *ku70*^*−/−*^ *bax*^*+/−*^ mice, the incidence of lymphoma was similar to that of *ku70*^*−/−*^ mice in both males (27.3%, *n*=22) and females (30.4%, *n*=23) (Supplementary Table S1), despite having an extended survival. Male *ku70*^*−/−*^ *bax*^*−/−*^ mice also had a similar incidence of lymphoma (25%, *n*=12) to *ku70*^*−/−*^ and *ku70*^*−/−*^ *bax*^*+/−*^ mice. However, 55.6% of the female *ku70*^*−/−*^ *bax*^*−/−*^ mice (*n*=9) developed lymphoma compared with *ku70*^*−/−*^ and *ku70*^*−/−*^ *bax*^*+/−*^ mice (Supplementary Table S1), but this increased incidence may be because of the longer lifespan observed in *ku70*^*−/−*^ *bax*^*−/−*^ females.

*Ku70*^*−/−*^ mice also developed rectal prolapse (12.5%) and abnormal teeth (19.7%) (Table 1). In the *ku70*^*−/−*^ mice with abnormal teeth, we observed malocclusion (misaligned teeth) in mice that died at a young age (*n*=3) (Table 1 and Supplementary Figure S2). Malocclusion at a young age, especially shortly after weaning, can impair the ability to eat, making these mice more prone to starvation and malnutrition (Supplementary Figure S2, see *ku70*^*−/−*^ mice (52 days)). In addition, we observed abnormally long teeth in adult *ku70*^*−/−*^ mice (*n*=8) that likely occurred as a result of a decrease in gnawing behavior due to other underlying illness. For example, one of the *ku70*^*−/−*^ mice (130 days old) (Supplementary Figure S2) was found to have severe lung necrosis and abnormal cardiac hypertrophy at the time of death. The incidence of abnormal teeth was lower in Bax-deficient Ku70-null mice (Table 1), and this difference may contribute to their improved survival. When we analyzed survival, excluding malocclusion, the median lifespan of Bax-deficient Ku70-null mice remained greater than *ku70*^*−/−*^ mice (Supplementary Figure S3). Therefore, it is likely that other health effects besides the decreased teeth impairment from Bax deficiency are contributing to enhanced survival.

Approximately half of all of the dead mutant mice were not analyzed for the cause of death because of bodily damage caused by other mice or excessive post-mortem necrosis. These mice are listed under ‘unknown cause of death' in Table 1. We suspect that some of these mice developed lymphoma or other diseases, including defects in the heart and lungs, which is discussed below. Therefore, the actual occurrence rate of these diseases may be higher than the rate that was confirmed by analyses of fresh samples. In addition, infectious diseases may also account for the death in the unknown group as seen in Ku80 (Lupus Ku autoantigen p80) knockout mice.^35^

### Aging-related changes detected by macroscopic analysis

Ku70-deficient mice develop age-associated changes, such as kyphosis and alopecia, earlier than normal.^6^ Interestingly, Bax deficiency delayed the development of some of these age-associated changes in Ku70-null mice (Supplementary Table S2). Furthermore, *ku70*^*−/−*^ mice older than 6 months old did not have visually detectable subcutaneous fat, consistent with the disappearance of subcutaneous fat reported in prematurely aging mice with mutant A-type lamins.^36, 37^ Bax-deficient Ku70-null mice of the same age maintained this fat layer (Supplementary Table S2). Altogether, these results suggest that the absence of Bax-mediated apoptosis can slow down the progression of organismal aging and loss of subcutaneous fat in Ku70-null mice.

### Ku70-null mice develop lung abnormalities

**Figure 4:**
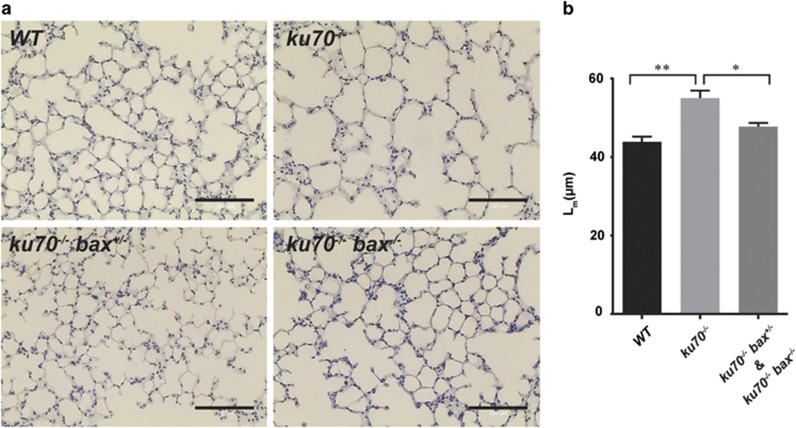
Emphysema was identified as a potential cause of premature death in *ku70*^*−/−*^ mice. (**a**) Representative lung tissue sections of 100-day-old mice show that *ku70*^*−/−*^ mice developed enlarged alveolar spaces, indicative of emphysema. Bax deficiency was able to delay the onset of emphysema in *Ku70-*null mice. (**b**) Calculations of mean length intercept (*L*_m_) were determined to measure the differences in alveolar sizes. *WT* (*n*=9), *ku70*^*−/−*^ (*n*=7), *ku70*^*−/−*^ *bax*^*+/−*^ (*n*=2), and *ku70*^*−/−*^ *bax*^*−/−*^ (*n*=2); *P<0.05, **P<0.001

**Figure 5:**
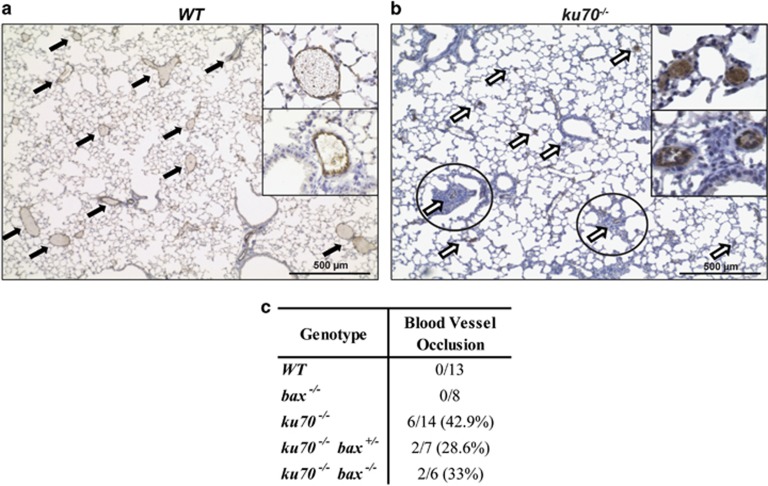
Blood vessel occlusions were found in the lungs of 100-day-old *ku70*^*−/−*^ mice. Immunohistochemical staining with the endothelial cell marker, von Willebrand Factor (vWF), in (**a**) *WT* and (B) *ku70*^*−/−*^ showed the presence of pulmonary arterial occlusions only in *ku70*^*−/−*^ mice. *Ku70*^*−/−*^ mice also developed plexiform-like lesions around the occluded vessel (circled). (**c**) The table summarizes the number of mice in each genetic background that developed blood vessel occlusion by 100 days of age

Structural and vascular abnormalities were detected in the lungs of *ku70*^*−/−*^ mice (Figures 4 and 5 and Supplementary Figures S4 and S7). The diseases found in the lungs include emphysema (Figure 4), pulmonary arterial occlusion (Figure 5), and interstitial lung disease (Supplementary Figure S7), all of which can induce hypoxia and contribute to heart failure. Emphysema is caused by excessive lung epithelial cell death that results in the enlargement of the alveolar space.^38, 39^ Ku70-deficient alveolar epithelial cells have been shown to be hypersensitive to DNA damages^40, 41^ caused by DNA synthesis errors and reactive oxygen species (ROS). Based on histological analyses and mean length intercept measurements in 3–4-month-old mice (Figure 4), *ku70*^*−/−*^ mice had significantly larger alveolar space than wild-type mice (Figure 4b). Interestingly, the size of the alveolar space in 3–4-month-old *ku70*^*−/−*^ *bax*^*+/−*^ and *ku70*^*−/−*^ *bax*^*−/−*^ mice were similar to that of wild type (Figure 4b), suggesting that the absence of Bax-mediated apoptosis can attenuate the development of emphysema in Ku70-null mice. The delayed onset of emphysema may be another factor in the improved survival rate in Bax-deficient Ku70-null mice. In fact, numerous TUNEL (terminal deoxynucleotidyl transferase dUTP nick end labeling)-positive cells were detected in a third of the *ku70*^*−/−*^ mice examined (*n*=6), whereas apoptotic cells were hardly detected in wild-type (*n*=8) and *ku70*^*−/−*^ *bax*^*−/−*^ mice (*n*=6) (Supplementary Figure S4). Because we were not able to detect an abnormally large number of dying pulmonary cells in the other two-thirds of the *ku70*^*−/−*^ mice, we speculate that apoptotic cells are hard to detect *in vivo*, unlike in cell culture experiments, since dead cells can be cleared by macrophages and neighboring cells, especially if the *ku70*^*−/−*^ mice were in the stages of developing lung diseases.

Pulmonary arterial occlusions can lead to pulmonary arterial hypertension (PAH). Blood vessel occlusions were detected by staining the pulmonary arteries with an endothelial cell marker, von Willebrand factor (vWF) (Figure 5).^42^ Occluded pulmonary arteries were observed in *ku70*^*−/−*^ mice and may be because of abnormal endothelial cell overgrowth (Figure 5b). Plexiform-like lesions surrounding the occluded blood vessels were also observed in *ku70*^*−/−*^ mice (Figure 5b, circled). Almost half of the *ku70*^*−/−*^ mice analyzed (42.9%) developed pulmonary vessel occlusions. However, Bax deficiency did not dramatically attenuate the incidence of these abnormalities (Figure 5c), suggesting that the extended survival of Bax-deficient Ku70-null mice was not mainly because of the prevention of PAH. Interestingly, a previous microarray gene expression study of lung tissue from patients with idiopathic PAH showed decreased Ku70 expression in 5 out of 6 patients.^43^ Our findings of pulmonary arterial occlusion in *ku70*^*−/−*^ mice could provide clues to help understand the mechanism underlying PAH.

Interstitial lung disease (ILD) was also observed in our mutant mice (Supplementary Figure S7). ILD is defined by an invasion of interstitial cells into the alveolar space and can result from different mechanisms, such as fibrosis, inflammation, or abnormal growth of lung epithelial cells.^44^ ILD was found more frequently in Bax-deficient Ku70-null mice than in *ku70*^*−/−*^ mice (Supplementary Figure S7D). However, ILD in these mice was not a result of fibrosis, excessive collagen deposition, or excessive smooth muscle growth, based on Masson's trichrome staining (Supplementary Figure S7A). In addition, ILD was not caused by inflammation or cancer as there was no accumulation of CD45-positive cells (a marker for leukocytes) or Ki67-positive cells, typical of dividing cancer cells (Supplementary Figure S7A–B). Type 1 and 2 alveolar epithelial cells were found in the regions of ILD (Supplementary Figure S7C), suggesting that this ILD, which destroyed alveolar structure, was caused by the abnormal growth and distribution of these cell types. ILD also progressed in an age-dependent manner as severe ILD was found more often in mice >6 months of age.

### Bax deficiency can attenuate cardiac defects in Ku70-null mice

**Figure 6:**
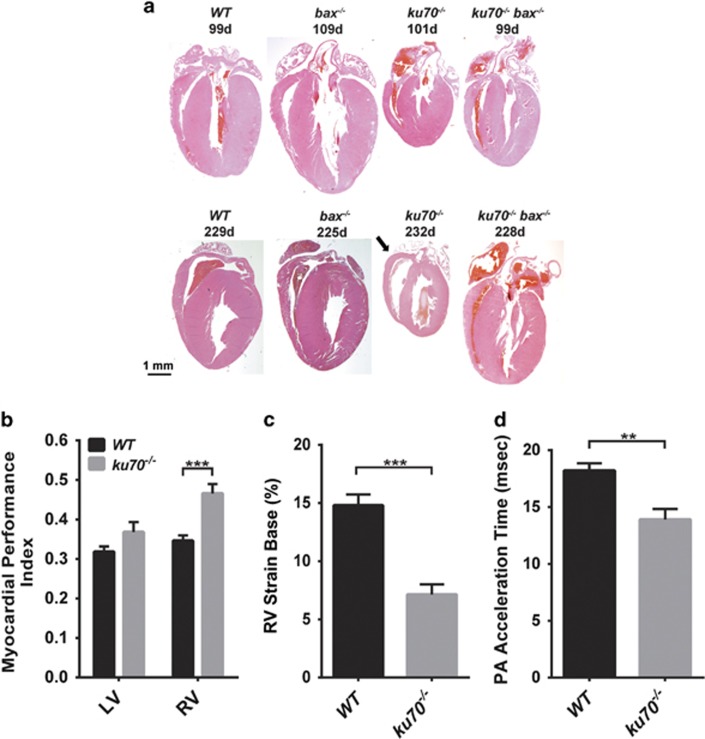
Cardiac histological and functional analyses. (**a**) Comparison of the hearts in a 4-chamber view is shown. H&E staining of representative hearts show structural abnormalities in 7-month-old *ku70*^*−/−*^ mice that have an enlarged right ventricle (RV) (arrow). The hearts of *ku70*^*−/−*^ mice were smaller than that of wild-type and other mutant mice. (**b–d**) Echocardiographic analyses of 4–6-month old *WT* (*n*=10) and *ku70*^*−/−*^ (*n*=10) mice show that *ku70*^*−/−*^ mice had (**b**) abnormal RV function, whereas left ventricular (LV) function is normal (*P*=0.1). A myocardial performance index (MPI) value >0.40 suggests that there is abnormal systolic and/or diastolic function. (**c**) Decreased strain in the basal region of the RV in *ku70*^*−/−*^ and (**d**) shorter pulmonary artery acceleration time suggest reduced muscle contractility and higher pressure load in the pulmonary arteries, respectively. **P<0.01, ***P<0.0001

Multiple signs of cardiac dysfunction were present in *ku70*^*−/−*^ mice. Enlargement of the right ventricle (RV) was observed in *ku70*^*−/−*^ mice by 7 months of age (Figure 6a; Supplementary Figure S6, arrows). Echocardiographic measurements of 4–6- month-old mice confirmed the presence of functional abnormalities in *ku70*^*−/−*^ mice (Figure 6b–d). The myocardial performance index (MPI), a measure of systolic and diastolic function,^45^ was determined for both the RV and left ventricle (LV), and values near 0.32 are considered normal. The MPI values for *ku70*^*−/−*^ mice were normal for the LV but was abnormal for the RV (Figure 6b). Longitudinal strain at the basal and mid-regions of the RV were measured to assess contractility.^34^ Ku70-null mice showed significantly lower absolute RV strain, and thus less contractility, compared with wild-type mice (Figure 6c). Furthermore, there was a greater pressure load in the pulmonary artery in *ku70*^*−/−*^ mice than in the wild-type mice, based on the shorter Doppler-determined acceleration time of blood flow through the pulmonary artery (Figure 6d). Our preliminary experiment showed that *ku70*^*−/−*^ *bax*^*−/−*^ mice had higher RV MPI than *wild-type* mice (in this preliminary experiment, all mice were analyzed on the same day under similar conditions, RV MPIs were 0.401±0.018 (wild type), 0.50±0.12 (*ku70*^*−/−*^), and 0.51±0.05 (*ku70*^*−/−*^*bax*^*−/−*^)). This observation suggests that Bax deficiency was not able to normalize heart function in *ku70*^*−/−*^ mice. However, this preliminary measurement was performed only once because of the difficulties with obtaining enough *ku70*^*−/−*^ and *ku70*^*−/−*^ *bax*^*−/−*^ mice for simultaneous comparative measurements. Therefore, further detailed analysis using *ku70*^*−/−*^ *bax*^*−/−*^ mice will be necessary to understand the mechanism of how Bax deletion can delay the onset of fatal heart failure in *ku70*^*−/−*^ mice.

Despite being slightly larger in body size than *ku70*^*−/−*^ mice (Figures 2a–c), *ku70*^*−/−*^ *bax*^*−/−*^ mice had disproportionately larger hearts (Figure 6a and Supplementary Figure S6). Based on histological analysis, the RVs in *ku70*^*−/−*^ *bax*^*−/−*^ mice were not as dilated compared with *ku70*^*−/−*^ mice (Figure 6a and Supplementary Figure S6), suggesting that the hearts of Bax-deficient Ku70-null mice may be able to tolerate stresses caused by the absence of Ku70 (e.g., DNA damage). We performed TUNEL staining, but we were not able to detect dying cells in the hearts collected from actively moving mice (Supplementary Figure S5). We speculate that, unlike in *in vitro* settings, the rapid clearance of apoptotic cells *in vivo* makes it difficult to detect the actual accumulation of dead cells.

### Bax deficiency did not reduce the DNA damage load in Ku70-null brains

Ku70 deficiency has been shown to decrease neuronal survival during development because of excessive neuronal apoptosis,^7^ whereas Bax deficiency has the opposite effect.^32^ When compared with wild-type mice, the overall brain size and weight of *ku70*^*−/−*^ mice were smaller whereas *bax*^*−/−*^ brains were larger (Figure 2e), and the number of hippocampal neurons showed a similar trend (Supplementary Figure S8A). Interestingly, *ku70*^*−/−*^ *bax*^*+/−*^ brains were only slightly heavier than *ku70*^*−/−*^ brains, even though *ku70*^*−/−*^ *bax*^*+/−*^ mice showed extended survival similar to the *ku70*^*−/−*^ *bax*^*−/−*^mice (Figures 1 and 2e). *Ku70*^*−/−*^ mice showed increased DNA DSBs in the hippocampus, based on phospho-*γ*H2A.X staining (Supplementary Figures S8B and C). The neurons in *ku70*^*−/−*^ *bax*^*+/−*^ and *ku70*^*−/−*^ *bax*^*−/−*^ mice were also positive for phospho-*γ*H2A.X; therefore, Bax deficiency did not help improve neuronal DNA damage repair despite its role in increasing the survival of these neurons.

### Bax deficiency in Ku70-null mice did not lead to abnormalities in the kidneys and liver

Unlike the lungs, heart, and brain, the kidneys and liver exhibited no obvious histological defects in both Ku70-null mice and Bax-deficient Ku70-null mice (Supplementary Figures S9 and S10). Based on Masson's trichrome staining, none of the mice analyzed developed liver fibrosis (Supplementary Figure S10A). The amount of DNA DSBs in the kidneys (Supplementary Figures S9A and D) and liver (Supplementary Figures S10B and E) at 3 months of age was similar across all genetic groups. Bax deficiency did not lead to excessive cell growth or tumorigenesis in the kidneys (Supplementary Figures S9B and E) and liver (Supplementary Figures S10C and F) based on Ki67 staining. Furthermore, to determine whether there were differences in the occurrence of apoptosis, cleaved Caspase-3 staining was performed (Supplementary Figures S9C and S10D). However, there are very few detectable Caspase 3-positive cells in these tissues, most likely because of the rapid clearance of apoptotic cells.

## Discussion

Previous studies have shown that Ku70 has anti-apoptotic activity by suppressing the intrinsic cell death signal mediated by Bax, in addition to its role in NHEJ DNA DSB repair.^8^ Our results support the hypothesis that the absence of Ku70 leads to Bax hyperactivation, giving rise to the development of degenerative diseases that culminate in an early death in *ku70*^*−/−*^ mice. Furthermore, the increased accumulation of DNA damage because of the absence of Ku70 can also trigger the DNA damage response to indirectly initiate apoptosis through p53-dependent Bax activation.^46^ We suspect that both of these mechanisms of Bax activation are contributing to the premature death observed in *ku70*^*−/−*^ mice.

In other previous studies, *ku70*^*−/−*^ *p53*^*−/−*^ and *ku80*^*−/−*^ *p53*^*−/−*^ mice developed tumors at much higher frequencies than single Ku70 or Ku80 knockout mice.^47, 48^ These observations indicate that NHEJ deficiency can cause oncogenic mutations and that p53-dependent cellular responses, such as cell cycle arrest and apoptosis, are important to suppress tumorigenesis. As p53 is intact in Bax-deficient Ku70-null mice, we speculate that p53 is able to suppress tumorigenesis caused by deficient NHEJ through p21-mediated cell cycle arrest and cellular senescence. Previous reports also show that cell cycle arrest, including cellular senescence, plays a greater role than apoptosis to suppress tumorigenesis in NHEJ-deficient mice.^49, 50^ Consistent with these findings, this study shows extended survival in male and female *ku70*^*−/−*^ *bax*^*+/−*^ mice and male *ku70*^*−/−*^ *bax*^*−/−*^ mice without a significant increase in the incidence of lymphoma (Supplementary Table S1). However, the higher incidence of lymphoma in female *ku70*^*−/−*^ *bax*^*−/−*^ mice may be reflective of the extended lifespan rather than an actual enhancement of tumorigenesis, as improved survival rate was more prominent in female than male *ku70*^*−/−*^ *bax*^*−/−*^ mice that showed no increased tumor incidence. These results further suggest that the partial suppression of Bax-mediated cell death, rather than complete suppression, may be more beneficial for the lifespan extension in Ku70-null mice without an increase in cancer risk.

Presently, it is unclear why Bax deficiency was able to enhance survival in Ku70-null mice more profoundly in females than males. Our results suggest that the effects caused by Bax activation in *ku70*^*−/−*^ mice seem to be more detrimental to females than males, based on the observation that survival is more significantly enhanced in females when Bax is deleted. The deletion of Bax has been shown to negatively impact males, causing testicular degeneration and infertility.^51^ Conversely, *bax*^*−/−*^ females maintain fertility longer in life because of increased oocyte survival,^52^ and this improvement in ovarian function may contribute to the better health span seen in Bax-deficient Ku70-null females to further enhance their survival. However, altered gonadal function does not fully explain the improved survival and extended lifespan observed in Ku70-null mice because Bax haploinsufficiency does not cause the same changes in gonadal function as the complete deletion of Bax, yet both gene mutations result in similar survival rates. *Bax*^*+/−*^ mice have functional ovaries and testes, and their reproduction is normal.^51^ Consistently, we observed successful mating and viable offspring from male and female *ku70*^*−/−*^ *bax*^*+/−*^ mice. Therefore, the influence of gonadal function is not the only reason how Bax deficiency can extend lifespan.

In comparison with *bax* haploinsufficiency (*bax*^*+/−*^), the complete deletion of *bax* (*bax*^*−/−*^) might have a greater protective influence on lung alveolar cells to suppress cell death caused by the absence of Ku70, and this might be why the maximum lifespan of *ku70*^*−/−*^ *bax*^*−/−*^ mice (951 days) was longer than that of *ku70*^*−/−*^*bax*^*+/−*^ mice (803 days) (Figure 1). However, the median survival of *ku70*^*−/−*^*bax*^*−/−*^ mice (38 weeks) and *ku70*^*−/−*^*bax*^*+/−*^ mice (37.5 weeks) did not show significant differences (Figure 1). The complete *bax* deletion is likely to enhance the survival of other cell types, such as the endothelial cells in the alveolar blood vessels, and this abnormality may cause a higher incidence of pulmonary blood vessel occlusion that we observed in the *ku70*^*−/−*^*bax*^*−/−*^ mice despite the increase in overall lifespan. In addition, *bax*^*−/−*^ mice, but not *bax*^*+/−*^ mice, have abnormally large brains because of increased neuronal survival during brain development^32^ that may have a negative impact on survival later in life. Furthermore, testicular development is suppressed in *bax*^*−/−*^ mice, but not in *bax*^*+/−*^ mice, and this leads to the absence of androgen synthesis in the testes.^51^ It is possible that the some of the benefits generated by Bax deletion in a Ku70-null background may be canceled by other inherent problems caused by the absence of Bax. In order to further examine the effects of Bax deletion, we are planning to conditionally knockout Bax in specific cell types, such as alveolar epithelial or endothelial cells, in a Ku70-null line.

In support of this study in which we have demonstrated a significant role of a pro-apoptosis gene in the induction of premature death in mutant mice with defects in DNA DSB repair, another report has shown that the deletion of PUMA, a BH3-only protein that activates Bax-mediated cell death, was able to prolong the survival of telomerase-defective mutant mice.^53^ Previous studies have shown that *ku70*^*−/−*^, *ku80*^*−/−*^, and *ku70*^*−/−*^ *ku80*^*−/−*^ mice exhibit similar abnormal aging phenotypes, including shortened lifespans.^6^ As Ku70 protein levels become very low in *ku80*^*−/−*^ cells,^6, 54^ *ku80*^*−/−*^ mice are expected to be phenotypically similar to *ku70*^*−/−*^ mice and have an increased DNA damage response as well as a lower threshold to initiate Bax-mediated apoptosis. The deletion of p21 was unable to extend the survival of *ku80*^*−/−*^ mice, despite being able to suppress cellular senescence in *ku80*^*−/−*^ MEFs.^55^ Importantly, this study shows that Bax deficiency can extend the survival and lifespan of *ku70*^*−/−*^ mice. Altogether, these observations suggest that apoptosis may potentially have a greater role than cellular senescence in lifespan determination, specifically in mice with defective NHEJ DNA repair.

This study also shows that Ku70 is essential to maintain normal lung alveolar structure and pulmonary arteries. Furthermore, this study suggests that Bax-mediated apoptosis plays a role in the development of emphysema in *ku70*^*−/−*^ mice. The hearts in aged *ku70*^*−/−*^ mice (>7 months old) also had functional and structural defects, suggesting that Ku70 is required for the maintenance of heart function in older mice. It is not well understood how the DNA damage response promotes the development of emphysema, pulmonary artery occlusion, and heart failure; therefore, further studies are clearly needed to understand the role of Ku70 in the maintenance of homeostasis in the pulmonary and cardiovascular systems.

## Materials and Methods

### Mouse husbandry and phenotypic observation

Genotyping for the Ku70 and Bax genes was performed as previously reported.^2, 51^ First, *ku70*^*+/−*^*bax*^*+/−*^ breeding pairs generated *ku70*^*+/+*^ *bax*^*+/+*^, *ku70*^*−/−*^ *bax*^*+/−*^, and *ku70*^*−/−*^ *bax*^*−/−*^ mice in order to analyze their age-associated disease phenotypes and overall lifespans. The housing and phenotypic observation of these mice were performed as previously reported in articles analyzing the abnormal aging phenotype in *ku70*^*−/−*^ mice.^6^ All mice were observed at least 6 times a week for the entire course of their lifespans. Moribund mice showing weight loss and decreased responsiveness were continually monitored multiple times a day, and all the mice were killed when they became immobile and could no longer reach the water source. Morbidities were scored by Kaplan–Meier analysis and measured for statistical significance by the log-rank test. The killed mice were observed by necropsy, and organs were removed and fixed for histology. All mice were housed in microisolator cages in a specific pathogen-free environment. The rodent diet was irradiated, and the bedding, wire top, isolator cage, cardholders, water, and water bottles were autoclaved. All mouse procedures were done in accordance with the Guide for the Care and Use of Laboratory Animals and approved by the institutional IACUC.

### Analysis of lung alveolar structure and size

Lungs were collected and fixed by intratracheal instillation of 4% paraformaldehyde to maintain the alveolar structure according to previously published methods.^52^ Tissue sections (7 *μ*m thick) of the upper left lobe were prepared for analysis. To determine the differences in alveolar size, measurements of mean linear intercept (*L*_m_) were used. *L*_m_ was calculated as previously described.^53, 54^ Pictures of at least 12 representative fields of lung sections were taken, avoiding large airways and blood vessels. Equidistant transverses of known length were superimposed on each picture and *L*_m_ was determined with the equation: *L*_m_=(*N*)(*L*)/*m*, where *N*=number of transverses in the image, *L*=length of each transverse, and *m*=sum of all intercepts. *L*_m_ was calculated for horizontal and vertical transverses, separately. For the horizontal transverses the values were *N*=22, *L*=461.25 *μ*m and for the vertical transverses, *N*=29, *L*=345.94 *μ*m.

### Immunohistochemistry

Harvested lungs were perfused, and hearts were incubated in a solution of 250 mM potassium chloride before fixation. All tissues were fixed overnight in 4% neutral buffered paraformaldehyde and then stored in sterile PBS at 4°C before being processed, embedded in paraffin, and sectioned (5–7 *μ*m). Sections were deparaffinized in xylene, rehydrated in a graded ethanol series, and incubated in a solution of 3% H_2_O_2_ in methanol for 20 min in order to quench endogenous peroxidase activity. Antigen retrieval was performed according to the manufacturer's instructions for the Biocare Medical Decloaking Chamber system (Concord, CA, USA). All sections were blocked for 1 h at room temperature before an overnight incubation in the primary antibody. The Vectastain Elite ABC Kit (Vector Laboratories, Burlingame, CA, USA; PK-6101) was used according to the manufacturer's instructions. Positive staining was visualized with DAB (ImmPACT DAB, Vector Laboratories, SK-4105). All sections were counterstained with hematoxylin for 30 s and dipped in acid alcohol as needed before being dehydrated and coverslipped. The following primary antibodies were used: phospho-*γ*-H2A.X (Cell Signaling Technologies, Danvers, MA, USA; no. 9718, 1 : 300), cleaved caspase-3 (Cell Signaling Technologies, Cambridge, MA, USA; no. 9664, 1 : 200), Ki67 (Abcam, Cambridge, MA, USA; ab15580, 1 : 200), *α*-smooth muscle actin (Abcam; ab5694, 1 : 5000) vWF (Dako, Carpenteria, CA, USA; A0082, 1 : 5000), CD45 (BD Pharmingen, San Diego, CA, USA; no. 550539, 1 : 50), T1*α* (Iowa Hybridoma Bank, Iowa City, IA, USA; clone 8.1.1, 5 *μ*g/ml), and pro-surfactant protein C (Millipore, Temecula, CA, USA; AB3786, 1 : 4000). Images were acquired using a Nikon Eclipse TE2000-S microscope (Melville, NY, USA) and the Metamorph software (Sunnyvale, CA, USA).

### Generation and culture of MEFs

MEF cultures were prepared from 14- to 15-day-old embryos from intercrossed *ku70*^*+/−*^ *bax*^*+/−*^ mice. After each embryo was removed and washed in PBS, the dark-colored visceral tissue from the abdominal region was removed. The remaining tissue was minced and digested in 0.25% trypsin for 20 min at 37°C in 5% CO_2_. The tissue was further broken down by pipetting before being plated onto a 10 cm^2^ dish in DMEM supplemented with 10% FBS, 1% non-essential amino acids, 1% (10 mM) L-glutamine, 1% (5 mM) sodium pyruvate, and 1% (50 U) of penicillin and streptomycin. At 80–90% confluency, the cells were passaged and plated in a 1 : 5 dilution. The cells were deemed MEFs once they acquired fibroblast cell morphology. The genotype of each embryo was confirmed with PCR as previously reported.^2, 51^

### Cell death assay

Wild-type and *ku70*^*−/−*^ MEFs were plated onto a 6-well plate at a density of 200 000 cells per well and treated with 1.0 *μ*M Doxorubicin (Sigma-Aldrch, St. Louis, MO, USA; 44583) for 24 h. Floating dead cells were collected, and the live cells were detached using 0.05% trypsin. After pelleting the cells and resuspending the cells to get a total volume of 100 *μ*l, the live and dead cells were counted manually based on Trypan blue dye exclusion. Apoptosis in tissue sections was detected by TUNEL staining using the *in situ* Apoptosis Detection Kit (4810-30-K, TAVS2 TdT-DAB; Trevigen, Gaithersburg, MD, USA).

### Cellular senescence assay

Wild-type and *ku70*^*−/−*^ MEFs were plated onto a 24-well plate at a density of 10 000 cells per well and cultured overnight. On the following day, cells were washed with warm (37°C) HBSS and then fixed with 4% neutral buffered paraformaldehyde for 10 min at room temperature. The senescence detection kit (Biovision, Milpitas, CA, USA; no. K320-250) was used according to the manufacturer's instructions to observe senescence-associated *β*-galactosidase activity in the MEFs.

### Western blotting

At 80–90% confluency, the floating dead cells were removed with HBSS. The remaining cells were scraped on ice, pelleted, and incubated in RIPA lysis buffer (150 mM NaCl, 1% NP-40, 0.5% sodium deoxycholate, and 0.1% SDS in PBS) containing a protease inhibitor cocktail (Thermo Scientific, Waltham, MA, USA; no. 87786) and PMSF for 20 min at 4°C. The total cell lysate was cleared via centrifugation at a speed of 14 000 r.p.m. (18 626 r.c.f.) for 30 min at 4°C. After determining the protein concentration using a Bradford assay (Bio-Rad, Hercules, CA, USA; no. 500-0006), 5 *μ*g of protein was prepared in Laemmli sample buffer, heat inactivated at 95°C for 5 min, separated using a 4–12% SDS-polyacrylamide gel (Life Technologies, Carlsbad, CA, USA, NuPAGE), and then transferred overnight onto a nitrocellulose membrane. The membranes were then dried at 65°C for 20 min before being blocked for 1 h at room temperature in 5% BSA, 3% milk, and 2% normal serum of the host species from which the secondary antibody was derived. The following primary antibodies were used: Ku70 N3H10 (NeoMarkers, Fremont, CA, USA; 1 : 500), Bax N20 (Santa Cruz Biotechnology, Inc., Dallas, TX, USA; sc-493, 1 : 1000), Caspase 3 (Cell Signaling; 1 : 1000), and *β*-actin (Sigma-Aldrich, A5441, 1 : 40 000). Horseradish peroxidase-conjugated goat anti-mouse (Life Technologies) or anti-rabbit IgG (Dako) was used as the secondary antibody. The incubation time for each primary antibody was 1 h at room temperature or overnight at 4°C, and the secondary antibodies were incubated for 1 h at room temperature. The bands were visualized using an ECL detection system (GE Healthcare, Pittsburgh, PA, USA; RPN2132).

### Echocardiography

Echocardiography was performed as previously described.^45^ Briefly, animals were anesthetized with 2% isoflurane supplemented with O_2_ in an isoflurane induction chamber and maintained with 1.5% isoflurane by nose cone. After depilating the chest, the extremities were secured to a warming pad (Braintree Scientific, Braintree, MA, USA) with paper tape, and needle electrodes were connected to a preamplifier to simultaneously record a single lead electrocardiogram. All image acquisitions and offline measurements were conducted by a single investigator who was blinded to animal groups. Image processing and data analysis were performed on the ultrasonograph using Syngo Vector Imaging technology software (Siemens Medical Solutions, Malvern, PA, USA).

### Statistical analyses

Statistical analyses were performed using GraphPad Prism for Windows, version 6.0 (GraphPad Software, San Diego, CA, USA). The log-rank test was used to compare the survival curves, and Student's *t*-test was used to compare the genotypes where appropriate.

## Figures and Tables

**Table 1:** Macroscopic abnormalities found in moribund/dead mice

**Genotype**	**Total**	**Lymphoma**			**Abnormalities found in:**			**Unknown cause of death**
		**Thymic**	**Disseminated**	**Total**	**Rectum**	**Teeth**		
						**Malocclusion**	**Long/thick teeth**	
*ku70*^*−/−*^	56	13	3	16 (28.6%)	7 (12.5%)	3 (5.4%)	8 (14.3%)	24 (42.9%)
*ku70*^*−/−*^ *bax*^*+/−*^	45	12	1	13 (28.9%)	4 (8.9%)	0	3 (6.7%)	27 (60%)
*ku70*^*−/−*^ *bax*^*−/−*^	21	8	0	8 (38.1%)	0	0	0	13 (61.9%)

## Abbreviations

Bax: Bcl-2-associated protein X
Ku70: Lupus Ku autoantigen p70
Ku80: Lupus Ku autoantigen p80
NHEJ: nonhomologous end joining
DSB: double-strand break
Apaf-1: apoptosis protease activation factor-1
MEF: mouse embryonic fibroblast
PAH: pulmonary artery hypertension
vWF: von Willebrand factor
ILD: interstitial lung disease
RV: right ventricle
LV: left ventricle
MPI: myocardial perform index
TUNEL: terminal deoxynucleotidyl transferase dUTP nick end labeling

## References

[bib1] Downs JA Jackson SP A means to a DNA end: the many roles of Ku Nat Rev Mol Cell Biol 2004 5 367 378 15122350 10.1038/nrm1367

[bib2] Gu Y Jin S Gao Y Weaver DT Alt FW Ku70-deficient embryonic stem cells have increased ionizing radiosensitivity, defective DNA end-binding activity, and inability to support V(D)J recombination Proc Natl Acad Sci USA 1997 94 8076 8081 9223317 10.1073/pnas.94.15.8076 PMC21559

[bib3] Gu Y Seidl KJ Rathbun GA Zhu C Manis JP van der Stoep N Growth retardation and leaky SCID phenotype of Ku70-deficient mice Immunity 1997 7 653 665 9390689 10.1016/s1074-7613(00)80386-6

[bib4] Li GC Ouyang H Li X Nagasawa H Little JB Chen DJ Ku70: a candidate tumor suppressor gene for murine T cell lymphoma Mol Cell 1998 2 1 8 9702186 10.1016/s1097-2765(00)80108-2

[bib5] Manis JP Gu Y Lansford R Sonoda E Ferrini R Davidson L Ku70 is required for late B cell development and immunoglobulin heavy chain class switching J Exp Med 1998 187 2081 2089 9625768 10.1084/jem.187.12.2081 PMC2212369

[bib6] Li H Vogel H Holcomb VB Gu Y Hasty P Deletion of Ku70, Ku80, or both causes early aging without substantially increased cancer Mol Cell Biol 2007 27 8205 8214 17875923 10.1128/MCB.00785-07 PMC2169178

[bib7] Gu Y Sekiguchi J Gao Y Dikkes P Frank K Ferguson D Defective embryonic neurogenesis in Ku-deficient but not DNA-dependent protein kinase catalytic subunit-deficient mice Proc Natl Acad Sci USA 2000 97 2668 2673 10716994 10.1073/pnas.97.6.2668 PMC15987

[bib8] Gomez JA Gama V Yoshida T Sun W Hayes P Leskov K Bax-inhibiting peptides derived from Ku70 and cell-penetrating pentapeptides Biochem Soc Trans 2007 35 (Pt 4 797 801 17635151 10.1042/BST0350797

[bib9] Gross A McDonnell JM Korsmeyer SJ BCL-2 family members and the mitochondria in apoptosis Genes Dev 1999 13 1899 1911 10444588 10.1101/gad.13.15.1899

[bib10] Wei MC Zong WX Cheng EH Lindsten T Panoutsakopoulou V Ross AJ Proapoptotic BAX and BAK: a requisite gateway to mitochondrial dysfunction and death Science 2001 292 727 730 11326099 10.1126/science.1059108 PMC3049805

[bib11] Kim SH Kim D Han JS Jeong CS Chung BS Kang CD Ku autoantigen affects the susceptibility to anticancer drugs Cancer Res 1999 59 4012 4017 10463600

[bib12] Gama V Gomez JA Mayo LD Jackson MW Danielpour D Song K Hdm2 is a ubiquitin ligase of Ku70-Akt promotes cell survival by inhibiting Hdm2-dependent Ku70 destabilization Cell Death Differ 2009 16 758 769 19247369 10.1038/cdd.2009.6 PMC2669846

[bib13] Cohen HY Lavu S Bitterman KJ Hekking B Imahiyerobo TA Miller C Acetylation of the C terminus of Ku70 by CBP and PCAF controls Bax-mediated apoptosis Mol Cell 2004 13 627 638 15023334 10.1016/s1097-2765(04)00094-2

[bib14] Cohen HY Miller C Bitterman KJ Wall NR Hekking B Kessler B Calorie restriction promotes mammalian cell survival by inducing the SIRT1 deacetylase Science 2004 305 390 392 15205477 10.1126/science.1099196

[bib15] Subramanian C Opipari AW Jr. Bian X Castle VP Kwok RP Ku70 acetylation mediates neuroblastoma cell death induced by histone deacetylase inhibitors Proc Natl Acad Sci USA 2005 102 4842 4847 15778293 10.1073/pnas.0408351102 PMC555706

[bib16] Li Y Yokota T Gama V Yoshida T Gomez JA Ishikawa K Bax-inhibiting peptide protects cells from polyglutamine toxicity caused by Ku70 acetylation Cell Death Differ 2007 14 2058 2067 17885668 10.1038/sj.cdd.4402219

[bib17] Mazumder S Plesca D Kinter M Almasan A Interaction of a cyclin E fragment with ku70 regulates bax-mediated apoptosis Mol Cell Biol 2007 27 3511 3520 17325036 10.1128/MCB.01448-06 PMC1899959

[bib18] Plesca D Mazumder S Gama V Matsuyama S Almasan A A C-terminal fragment of cyclin E, generated by caspase-mediated cleavage, is degraded in the absence of a recognizable phosphodegron J Biol Chem 2008 283 30796 30803 18784078 10.1074/jbc.M804642200 PMC2576529

[bib19] Iijima K Muranaka C Kobayashi J Sakamoto S Komatsu K Matsuura S NBS1 regulates a novel apoptotic pathway through Bax activation DNA Repair (Amst) 2008 7 1705 1716 18644472 10.1016/j.dnarep.2008.06.013

[bib20] Anekonda TS Adamus G Resveratrol prevents antibody-induced apoptotic death of retinal cells through upregulation of Sirt1 and Ku70 BMC Res Notes 2008 1 122 19046449 10.1186/1756-0500-1-122 PMC2633309

[bib21] Pucci S Mazzarelli P Sesti F Boothman DA Spagnoli LG Interleukin-6 affects cell death escaping mechanisms acting on Bax-Ku70-Clusterin interactions in human colon cancer progression Cell Cycle 2009 8 473 481 19177010 10.4161/cc.8.3.7652 PMC2853871

[bib22] Sundaresan NR Samant SA Pillai VB Rajamohan SB Gupta MP SIRT3 is a stress-responsive deacetylase in cardiomyocytes that protects cells from stress-mediated cell death by deacetylation of Ku70 Mol Cell Biol 2008 28 6384 6401 18710944 10.1128/MCB.00426-08 PMC2577434

[bib23] Trougakos IP Lourda M Antonelou MH Kletsas D Gorgoulis VG Papassideri IS Intracellular clusterin inhibits mitochondrial apoptosis by suppressing p53-activating stress signals and stabilizing the cytosolic Ku70-Bax protein complex Clin Cancer Res 2009 15 48 59 19118032 10.1158/1078-0432.CCR-08-1805 PMC4483278

[bib24] Yamaguchi H Woods NT Piluso LG Lee HH Chen J Bhalla KN p53 acetylation is crucial for its transcription-independent proapoptotic functions J Biol Chem 2009 284 11171 11183 19265193 10.1074/jbc.M809268200 PMC2670122

[bib25] Vishnudas VK Miller JB Ku70 regulates Bax-mediated pathogenesis in laminin-alpha2-deficient human muscle cells and mouse models of congenital muscular dystrophy Hum Mol Genet 2009 18 4467 4477 19692349 10.1093/hmg/ddp399 PMC2773263

[bib26] Zou H Volonte D Galbiati F Interaction of caveolin-1 with Ku70 inhibits Bax-mediated apoptosis PLoS One 2012 7 e39379 22745744 10.1371/journal.pone.0039379 PMC3380016

[bib27] Dumitru R Gama V Fagan BM Bower JJ Swahari V Pevny LH Human embryonic stem cells have constitutively active Bax at the Golgi and are primed to undergo rapid apoptosis Mol Cell 2012 46 573 583 22560721 10.1016/j.molcel.2012.04.002 PMC3372694

[bib28] Li JJ Gu QH Li M Yang HP Cao LM Hu CP Role of Ku70 and Bax in epigallocatechin-3-gallate-induced apoptosis of A549 cells *in vivo* Oncol Lett 2013 5 101 106 23255902 10.3892/ol.2012.972 PMC3525449

[bib29] Wang B Xie M Li R Owonikoko TK Ramalingam SS Khuri FR Role of Ku70 in deubiquitination of Mcl-1 and suppression of apoptosis Cell Death Differ 2014 21 1160 1169 24769731 10.1038/cdd.2014.42 PMC4207484

[bib30] De Zio D Bordi M Tino E Lanzuolo C Ferraro E Mora E The DNA repair complex Ku70/86 modulates Apaf1 expression upon DNA damage Cell Death Differ 2011 18 516 527 20966962 10.1038/cdd.2010.125 PMC3132004

[bib31] Harper JM Wilkinson JE Miller RA Macrophage migration inhibitory factor-knockout mice are long lived and respond to caloric restriction FASEB J 2010 24 2436 2442 20219983 10.1096/fj.09-152223 PMC2887269

[bib32] White FA Keller-Peck CR Knudson CM Korsmeyer SJ Snider WD Widespread elimination of naturally occurring neuronal death in Bax-deficient mice J Neurosci 1998 18 1428 1439 9454852 10.1523/JNEUROSCI.18-04-01428.1998 PMC6792725

[bib33] Gama V Yoshida T Gomez JA Basile DP Mayo LD Haas AL Involvement of the ubiquitin pathway in decreasing Ku70 levels in response to drug-induced apoptosis Exp Cell Res 2006 312 488 499 16368436 10.1016/j.yexcr.2005.11.016

[bib34] Azam S Desjardins CL Schluchter M Liner A Stelzer JE Yu X Comparison of velocity vector imaging echocardiography with magnetic resonance imaging in mouse models of cardiomyopathy Circ Cardiovasc Imaging 2012 5 776 781 22977126 10.1161/CIRCIMAGING.111.972406 PMC3504170

[bib35] Vogel H Lim DS Karsenty G Finegold M Hasty P Deletion of Ku86 causes early onset of senescence in mice Proc Natl Acad Sci USA 1999 96 10770 10775 10485901 10.1073/pnas.96.19.10770 PMC17958

[bib36] Mounkes LC Stewart CL Aging and nuclear organization: lamins and progeria Curr Opin Cell Biol 2004 16 322 327 15145358 10.1016/j.ceb.2004.03.009

[bib37] Mounkes LC Kozlov S Hernandez L Sullivan T Stewart CL A progeroid syndrome in mice is caused by defects in A-type lamins Nature 2003 423 298 301 12748643 10.1038/nature01631

[bib38] Thurlbeck WM Muller NL Emphysema: definition, imaging, and quantification AJR Am J Roentgenol 1994 163 1017 1025 7976869 10.2214/ajr.163.5.7976869

[bib39] Turato G Zuin R Saetta M Pathogenesis and pathology of COPD Respiration 2001 68 117 128 11287822 10.1159/000050478

[bib40] Koike M Yutoku Y Koike A The defect of Ku70 affects sensitivity to X-ray and radiation-induced caspase-dependent apoptosis in lung cells J Vet Med Sci 2012 75 415 420 23149547 10.1292/jvms.12-0333

[bib41] Koike M Yutoku Y Koike A Accumulation of Ku70 at DNA double-strand breaks in living epithelial cells Exp Cell Res 2011 317 2429 2437 21820429 10.1016/j.yexcr.2011.07.018

[bib42] Nicolls MR Mizuno S Taraseviciene-Stewart L Farkas L Drake JI Al Husseini A New models of pulmonary hypertension based on VEGF receptor blockade-induced endothelial cell apoptosis Pulm Circ 2012 2 434 442 23372927 10.4103/2045-8932.105031 PMC3555413

[bib43] Voelkel NF Gomez-Arroyo J Abbate A Bogaard HJ Nicolls MR Pathobiology of pulmonary arterial hypertension and right ventricular failure Eur Respir J 2012 40 1555 1565 22743666 10.1183/09031936.00046612 PMC4019748

[bib44] Bourke SJ Interstitial lung disease: progress and problems Postgrad Med J 2006 82 494 499 16891438 10.1136/pgmj.2006.046417 PMC2585700

[bib45] Morgan EE Faulx MD McElfresh TA Kung TA Zawaneh MS Stanley WC Validation of echocardiographic methods for assessing left ventricular dysfunction in rats with myocardial infarction Am J Physiol Heart Circ Physiol 2004 287 H2049 H2053 15475530 10.1152/ajpheart.00393.2004

[bib46] Miyashita T Reed JC Tumor suppressor p53 is a direct transcriptional activator of the human bax gene Cell 1995 80 293 299 7834749 10.1016/0092-8674(95)90412-3

[bib47] Holcomb VB Vogel H Marple T Kornegay RW Hasty P Ku80 and p53 suppress medulloblastoma that arise independent of Rag-1-induced DSBs Oncogene 2006 25 7159 7165 16751807 10.1038/sj.onc.1209704

[bib48] Li H Choi YJ Hanes MA Marple T Vogel H Hasty P Deleting Ku70 is milder than deleting Ku80 in p53-mutant mice and cells Oncogene 2009 28 1875 1878 19330025 10.1038/onc.2009.57

[bib49] Van Nguyen T Puebla-Osorio N Pang H Dujka ME Zhu C DNA damage-induced cellular senescence is sufficient to suppress tumorigenesis: a mouse model J Exp Med 2007 204 1453 1461 17535972 10.1084/jem.20062453 PMC2118600

[bib50] Foster SS De S Johnson LK Petrini JH Stracker TH Cell cycle- and DNA repair pathway-specific effects of apoptosis on tumor suppression Proc Natl Acad Sci USA 2012 109 9953 9958 22670056 10.1073/pnas.1120476109 PMC3382548

[bib51] Knudson CM Tung KS Tourtellotte WG Brown GA Korsmeyer SJ Bax-deficient mice with lymphoid hyperplasia and male germ cell death Science 1995 270 96 99 7569956 10.1126/science.270.5233.96

[bib52] Perez GI Jurisicova A Wise L Lipina T Kanisek M Bechard A Absence of the proapoptotic Bax protein extends fertility and alleviates age-related health complications in female mice Proc Natl Acad Sci USA 2007 104 5229 5234 17360389 10.1073/pnas.0608557104 PMC1817832

[bib53] Sperka T Song Z Morita Y Nalapareddy K Guachalla LM Lechel A Puma and p21 represent cooperating checkpoints limiting self-renewal and chromosomal instability of somatic stem cells in response to telomere dysfunction Nat Cell Biol 2012 14 73 79 22138576 10.1038/ncb2388

[bib54] Koike M Koike A The Ku70-binding site of Ku80 is required for the stabilization of Ku70 in the cytoplasm, for the nuclear translocation of Ku80, and for Ku80-dependent DNA repair Exp Cell Res 2005 305 266 276 15817152 10.1016/j.yexcr.2004.12.027

[bib55] Zhao B Benson EK Qiao R Wang X Kim S Manfredi JJ Cellular senescence and organismal ageing in the absence of p21(CIP1/WAF1) in ku80(−/−) mice EMBO Rep 2009 10 71 78 19079133 10.1038/embor.2008.220 PMC2613205

