# Supplementary material for: Bax deficiency extends the survival of Ku70 knockout mice that develop lung and heart diseases
Source: Cell Death Dis. 2015 Mar 26;6(3):e1706–. doi: 10.1038/cddis.2015.11 (PMC4385910; doi:10.1038/cddis.2015.11)
Supplement: Supplementary Table S1 [file cddis201511x1.pdf]

Supplementary Figures

Supplementary Table 1

Age and Sex-Specific Fatalities due to Lymphoma									
**Mouse Age (weeks)**	***ku70*** $^{-/-}$			***ku70*** $^{-/-}$ ***bax*** $^{+/-}$			***ku70*** $^{-/-}$ ***bax*** $^{-/-}$		
	**Females**	**Males**	**Total**	**Females**	**Males**	**Total**	**Females**	**Males**	**Total**
**3-8**	0/5	0/6	0/11	0/0	0/2	0/2	0/0	0/1	0/1
**9-12**	0/1	0/0	0/1	0/0	0/1	0/1	0/0	0/0	0/0
**13-25**	3/6 (50%)	0/9	3/15 (20%)	0/2	0/4	0/6	0/1	0/3	0/4
**>25**	4/9 (44.4%)	9/18 (50%)	13/27 (48.1%)	7/21 (33.3%)	6/15 (40%)	13/36 (36.1%)	5/8 (62.5%)	3/8 (37.5%)	8/16 (50%)
**Total**	7/21 (33.3%)	9/33 (27.3%)	16/54 (29.6%)	7/23 (30.4%)	6/22 (27.3%)	13/45 (28.9%)	5/9 (55.6%)	3/12 (25%)	8/21 (38.1%)
