# Supplementary material for: Bax deficiency extends the survival of Ku70 knockout mice that develop lung and heart diseases
Source: Cell Death Dis. 2015 Mar 26;6(3):e1706–. doi: 10.1038/cddis.2015.11 (PMC4385910; doi:10.1038/cddis.2015.11)
Supplement: Supplementary Table S2 [file cddis201511x2.pdf]

Supplementary Table 2

Age-Associated Characteristics				
**Mouse Age (weeks)**	**Genotype**	**Kyphosis**	**Alopecia**	**Absence of Subcutaneous Fat**
**3-20**	*WT*	0/22	0/22	2/18 (11.1%)
	ku70 $^{-/-}$	2/14 (14.3%)	1/17 (5.9%)	12/13 (92.3%)
	ku70 $^{-/-}$ *bax* $^{+/-}$	0/5	0/5	1/4 (25%)
	ku70 $^{-/-}$ *bax* $^{-/-}$	0/6	0/6	0/4
**21-30**	*WT*	0/9	0/9	0/9
	ku70 $^{-/-}$	2/12 (16.7%)	1/12 (8.3%)	10/10 (100%)
	ku70 $^{-/-}$ *bax* $^{+/-}$	0/6	1/6 (16.7%)	1/4 (25%)
	ku70 $^{-/-}$ *bax* $^{-/-}$	0/4	0/4	1/2 (50%)
**31-50**	*WT*	0/13	0/12	0/12
	ku70 $^{-/-}$	1/13 (7.7%)	1/13 (7.7%)	11/11 (100%)
	ku70 $^{-/-}$ *bax* $^{+/-}$	4/25 (16%)	0/27	5/18 (27.8%)
	ku70 $^{-/-}$ *bax* $^{-/-}$	0/7	0/7	3/7 (42.9%)
**>51**	*WT*	3/41 (7.3%)	11/42 (26.2%)	9/41 (22%)
	ku70 $^{-/-}$	2/4 (50%)	3/4 (75%)	4/4 (100%)
	ku70 $^{-/-}$ *bax* $^{+/-}$	4/6 (66.7%)	3/6 (50%)	3/6 (50%)
	ku70 $^{-/-}$ *bax* $^{-/-}$	1/4 (25%)	1/4 (25%)	1/2 (50%)
**Total**	*WT*	3/85 (3.5%)	11/85 (12.9%)	11/80 (13.8%)
	ku70 $^{-/-}$	7/43 (16.3%)	6/46 (13%)	37/38 (97.4%)
	ku70 $^{-/-}$ *bax* $^{+/-}$	8/42 (19%)	4/44 (9.1%)	10/32 (31.3%)
	ku70 $^{-/-}$ *bax* $^{-/-}$	1/21 (4.8%)	1/21 (4.8%)	5/15 (33.3%)
