# Supplementary material for: Bax deficiency extends the survival of Ku70 knockout mice that develop lung and heart diseases
Source: Cell Death Dis. 2015 Mar 26;6(3):e1706–. doi: 10.1038/cddis.2015.11 (PMC4385910; doi:10.1038/cddis.2015.11)
Supplement: Supplementary Figure S1 [file cddis201511x3.pdf]

Figure S1

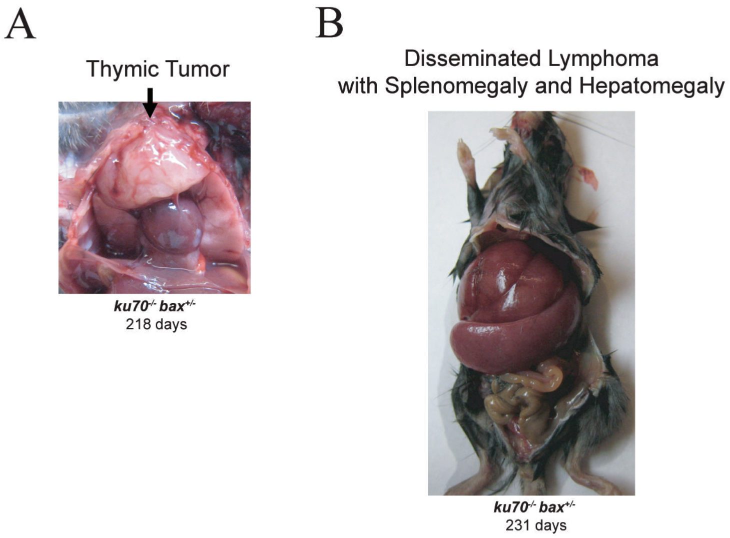

Figure S1. Examples of a (A) thymic tumor and (B) disseminated lymphoma with splenomegaly and hepatomegaly.
