# Supplementary material for: Bax deficiency extends the survival of Ku70 knockout mice that develop lung and heart diseases
Source: Cell Death Dis. 2015 Mar 26;6(3):e1706–. doi: 10.1038/cddis.2015.11 (PMC4385910; doi:10.1038/cddis.2015.11)
Supplement: Supplementary Figure S2 [file cddis201511x4.pdf]

Figure S2

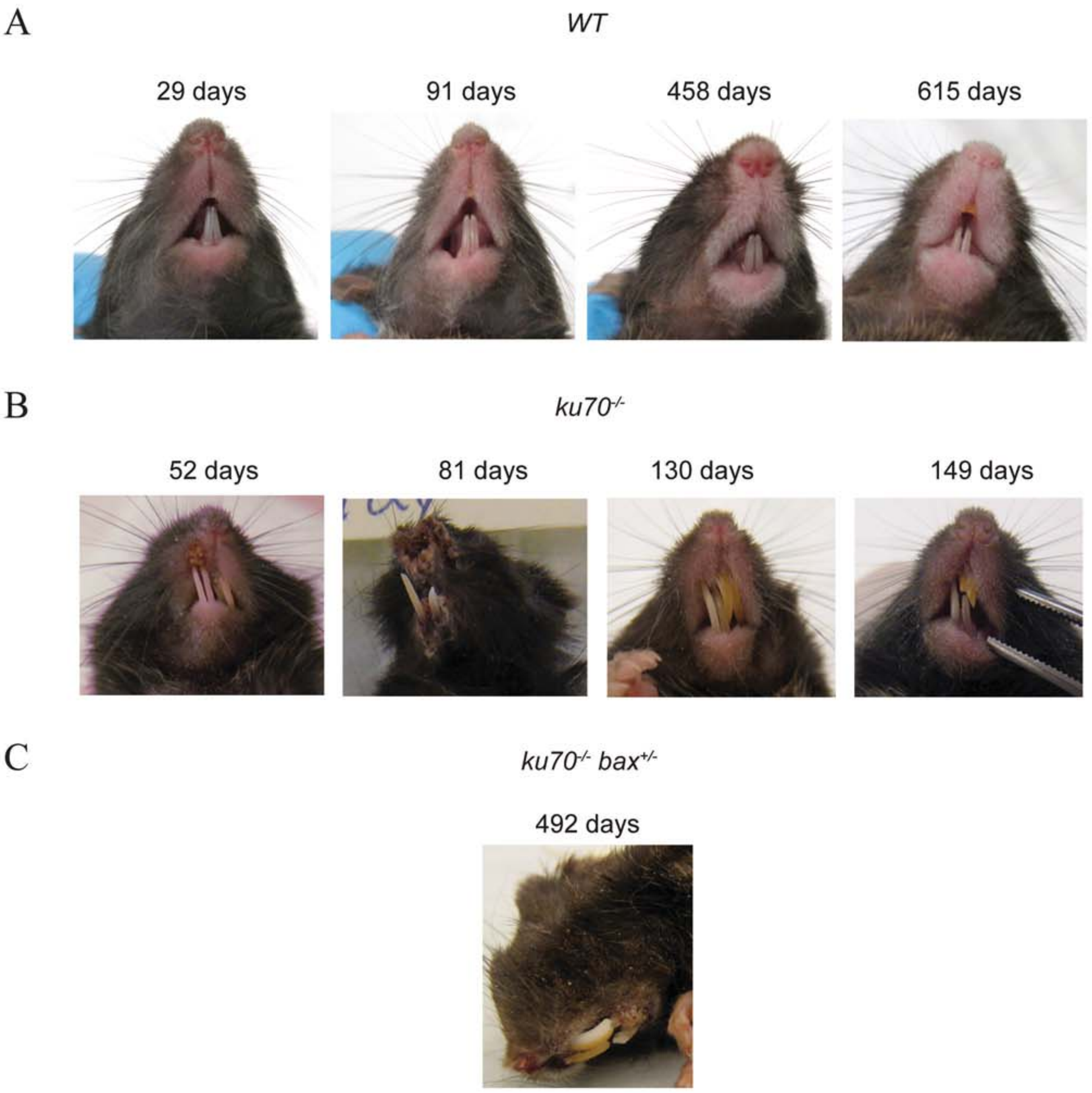

Figure S2. Abnormal teeth growth was detected in *ku70*$^{-/-}$ mice. Normal teeth in *WT* mice and abnormal teeth in *ku70*$^{-/-}$ mice are shown.
