# Supplementary material for: Bax deficiency extends the survival of Ku70 knockout mice that develop lung and heart diseases
Source: Cell Death Dis. 2015 Mar 26;6(3):e1706–. doi: 10.1038/cddis.2015.11 (PMC4385910; doi:10.1038/cddis.2015.11)
Supplement: Supplementary Figure S3 [file cddis201511x5.pdf]

Figure S3

A

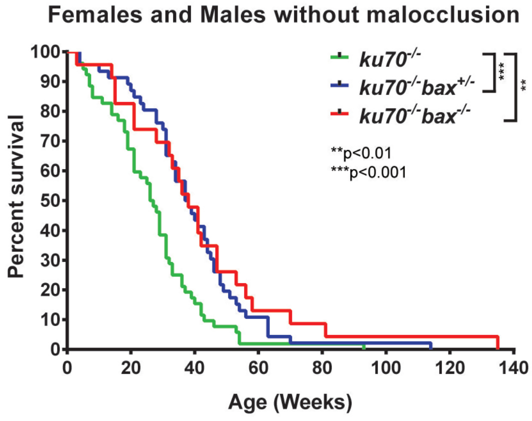

B

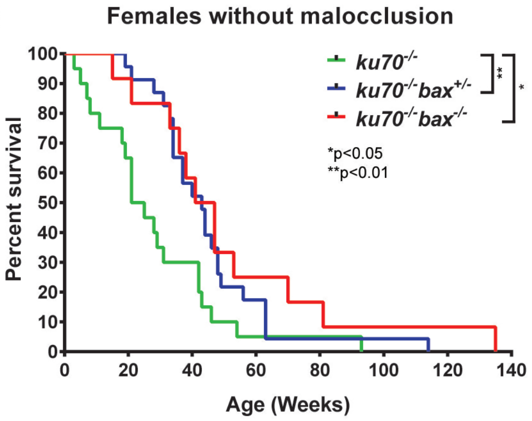

C

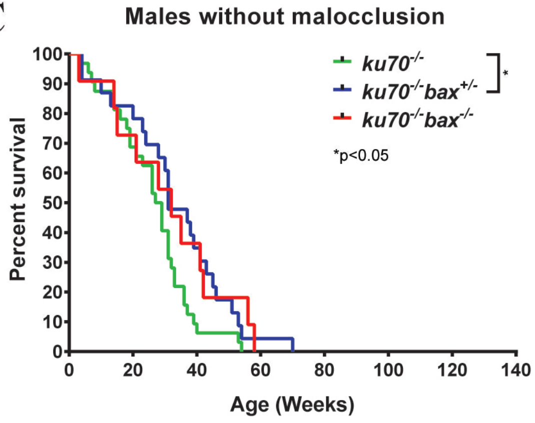

D

Median Survival (weeks)			
**Genotype**	**Total**	**Females**	**Males**
ku70 $^{-/-}$	26.5 (n=52)	23 (n=20)	28 (n=32)
ku70 $^{-/-}$ ***bax*** $^{+/-}$	37.5 (n=46)	43 (n=23)	31 (n=23)
ku70 $^{-/-}$ ***bax*** $^{-/-}$	38 (n=23)	44 (n=12)	32 (n=11)

Figure S3. Survival of Bax-deficient Ku70 null mice, excluding mice with malocclusion (misaligned teeth). Kaplan-Meier survival curves are shown for (A) all mice analyzed, (B) females and (C) males. (D) The table summarizes the median survival of all mice analyzed.
