# Supplementary figures and images for: Bax deficiency extends the survival of Ku70 knockout mice that develop lung and heart diseases
Source: Cell Death Dis. 2015 Mar 26;6(3):e1706–. doi: 10.1038/cddis.2015.11 (PMC4385910; doi:10.1038/cddis.2015.11)

Figure S4

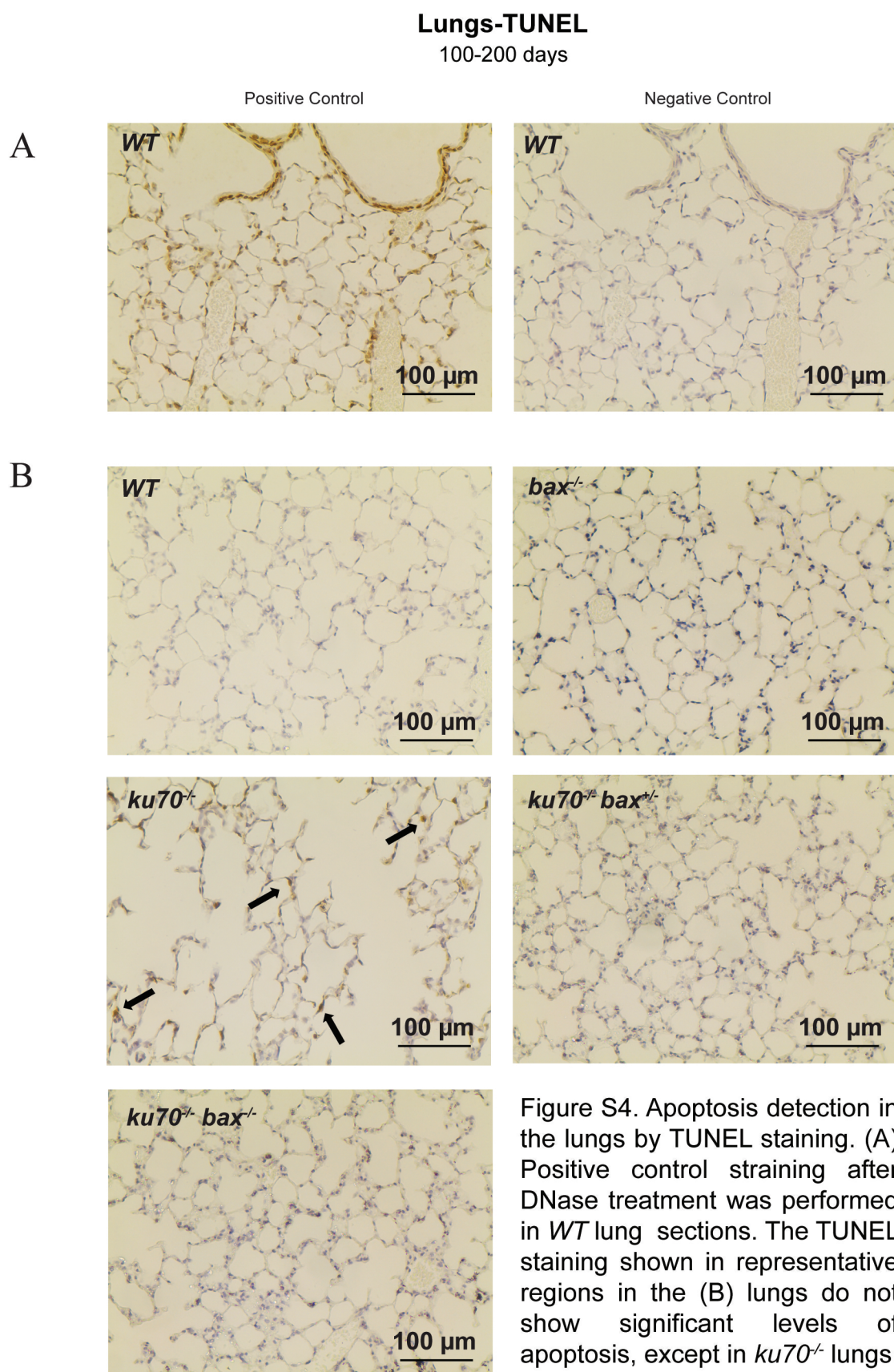

Supplement: Supplementary Figure S4 [file cddis201511x6.pdf]
