# Supplementary material for: Bax deficiency extends the survival of Ku70 knockout mice that develop lung and heart diseases
Source: Cell Death Dis. 2015 Mar 26;6(3):e1706–. doi: 10.1038/cddis.2015.11 (PMC4385910; doi:10.1038/cddis.2015.11)
Supplement: Supplementary Figure S5 [file cddis201511x7.pdf]

Figure S5

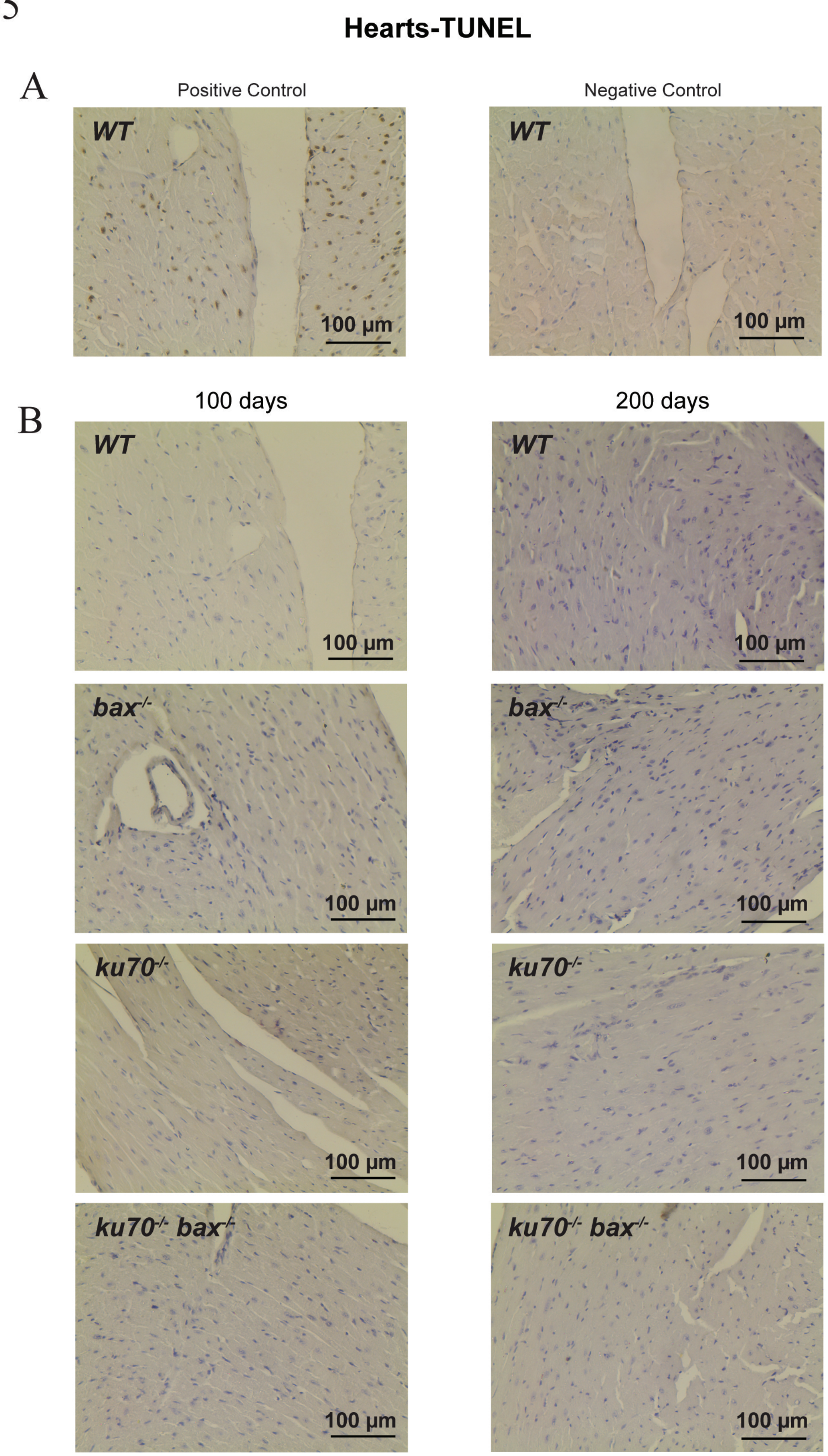

Figure S5. Apoptosis detection in the heart by TUNEL staining. (A) Positive control straining after DNase treatment was performed in *WT* heart sections. The TUNEL staining shown in representative regions in the (B) hearts do not show significant levels of apoptosis.
