# Supplementary material for: Bax deficiency extends the survival of Ku70 knockout mice that develop lung and heart diseases
Source: Cell Death Dis. 2015 Mar 26;6(3):e1706–. doi: 10.1038/cddis.2015.11 (PMC4385910; doi:10.1038/cddis.2015.11)
Supplement: Supplementary Figure S6 [file cddis201511x8.pdf]

Figure S6

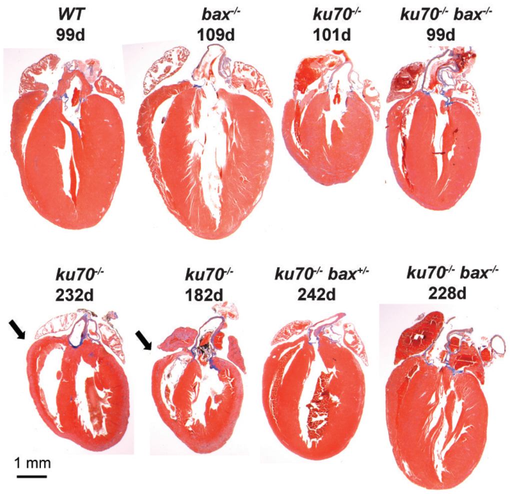

Figure S6. Masson's trichrome staining of heart sections showed no significantly detectable fibrosis (blue staining indicative of collagen deposition) within the cardiac chambers of 100 and 200 day old mice. A comparison of the hearts in a 4-chamber view is shown. Only the hearts from *ku70*$^{-/-}$ mice have enlarged right ventricles (arrow), a symptom of pulmonary hypertension.
