# Supplementary material for: Bax deficiency extends the survival of Ku70 knockout mice that develop lung and heart diseases
Source: Cell Death Dis. 2015 Mar 26;6(3):e1706–. doi: 10.1038/cddis.2015.11 (PMC4385910; doi:10.1038/cddis.2015.11)
Supplement: Supplementary Figure S7 [file cddis201511x9.pdf]

Figure S7

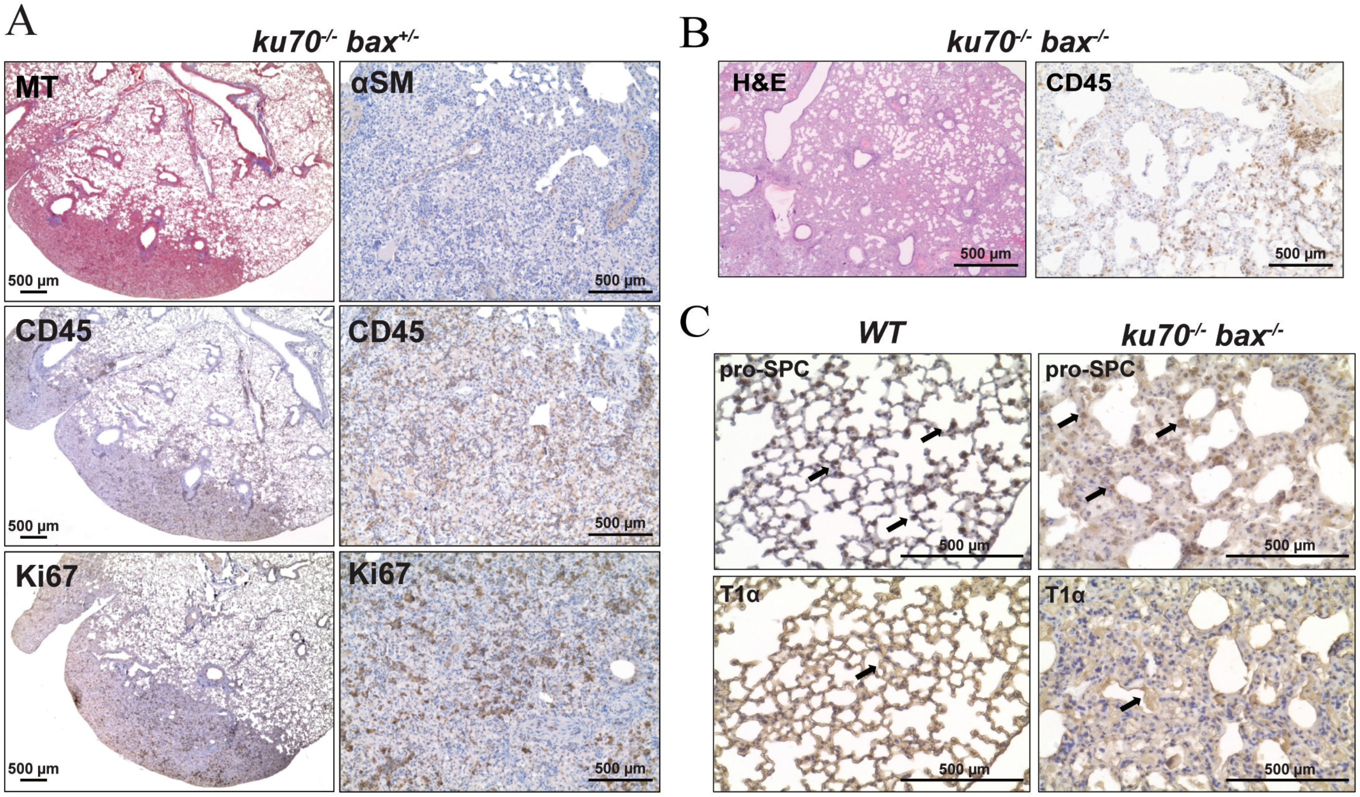

D

Genotype	Interstitial Lung Disease
WT	0/14
bax $^{-/-}$	0/12
ku70 $^{-/-}$	2/15 (13.3%)
ku70 $^{-/-}$ ***bax*** $^{+/-}$	2/9 (22.2%)
ku70 $^{-/-}$ ***bax*** $^{-/-}$	4/8 (50%)

Figure S7. Interstitial lung disease (ILD) was observed in Bax-deficient Ku70 null mice. Immunohistochemical analyses showed that ILD was not the result of fibrosis or the abnormal growth of smooth muscle cells as detected by (A) Masson's trichrome (MT) staining and α-smooth muscle actin (αSM), respectively. Based on the immunostaining patterns of (A) Ki67 and (A)-(B) CD45, the areas of ILD were not caused by cancer or inflammation due to the infiltration of CD45+ cells, respectively (C) Type II (pro-surfactant C) and Type I (T1α) lung alveolar epithelial cells immunostaining show that there was excessive cell growth and distribution of these cells in the areas of ILD. (D) The table summarizes the number of mice in each genetic background that developed interstitial lung disease, which was determined by the presence of multiple alveolar epithelial cell layers.
