# Supplementary material for: Bax deficiency extends the survival of Ku70 knockout mice that develop lung and heart diseases
Source: Cell Death Dis. 2015 Mar 26;6(3):e1706–. doi: 10.1038/cddis.2015.11 (PMC4385910; doi:10.1038/cddis.2015.11)
Supplement: Supplementary Figure S8 [file cddis201511x10.pdf]

Figure S8

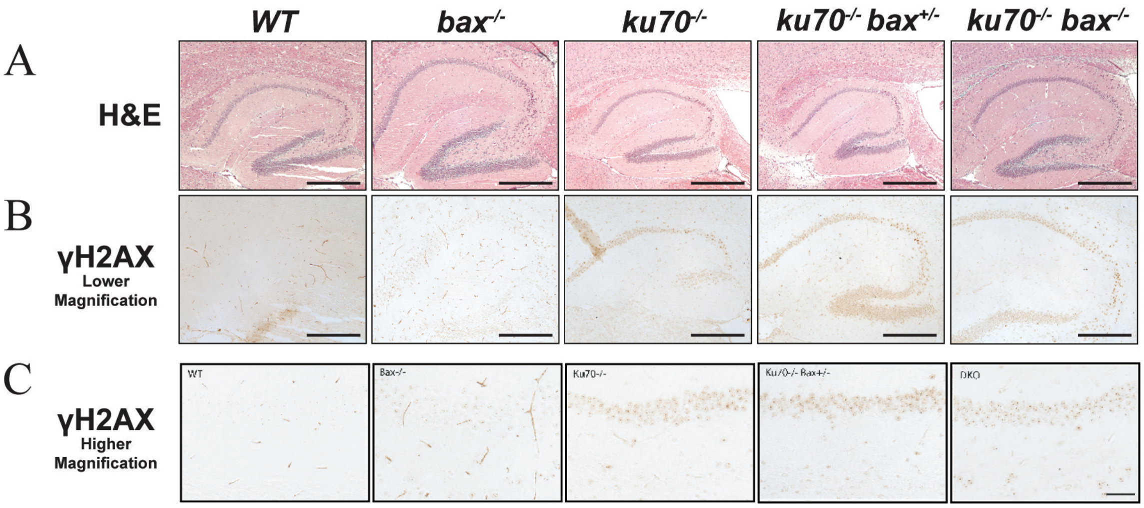

Figure S8. Bax deficiency may improve neuronal survival in the absence of Ku70. (A) Comparison of sagittal brain sections is shown. Despite the smaller overall body weight of Bax-deficient Ku70 null mice, the area of the hippocampus in *ku70*$^{-/-}$ *bax*$^{-/-}$ brains was similar in size to *WT*. (B) Hippocampal staining for phospho-γ-H2AX, a marker for DNA DSBs, showed that the neurons in Bax-deficient mice accrue DNA damage in the absence of Ku70, but these neurons are still present and able to survive. The scale bar in (A) and (B) represents 500 μm and 100 μm in (C). (C) Higher magnification of phospho-γ-H2AX staining in the hippocampus.
