# Supplementary material for: Bax deficiency extends the survival of Ku70 knockout mice that develop lung and heart diseases
Source: Cell Death Dis. 2015 Mar 26;6(3):e1706–. doi: 10.1038/cddis.2015.11 (PMC4385910; doi:10.1038/cddis.2015.11)
Supplement: Supplementary Figure S9 [file cddis201511x11.pdf]

Figure S9

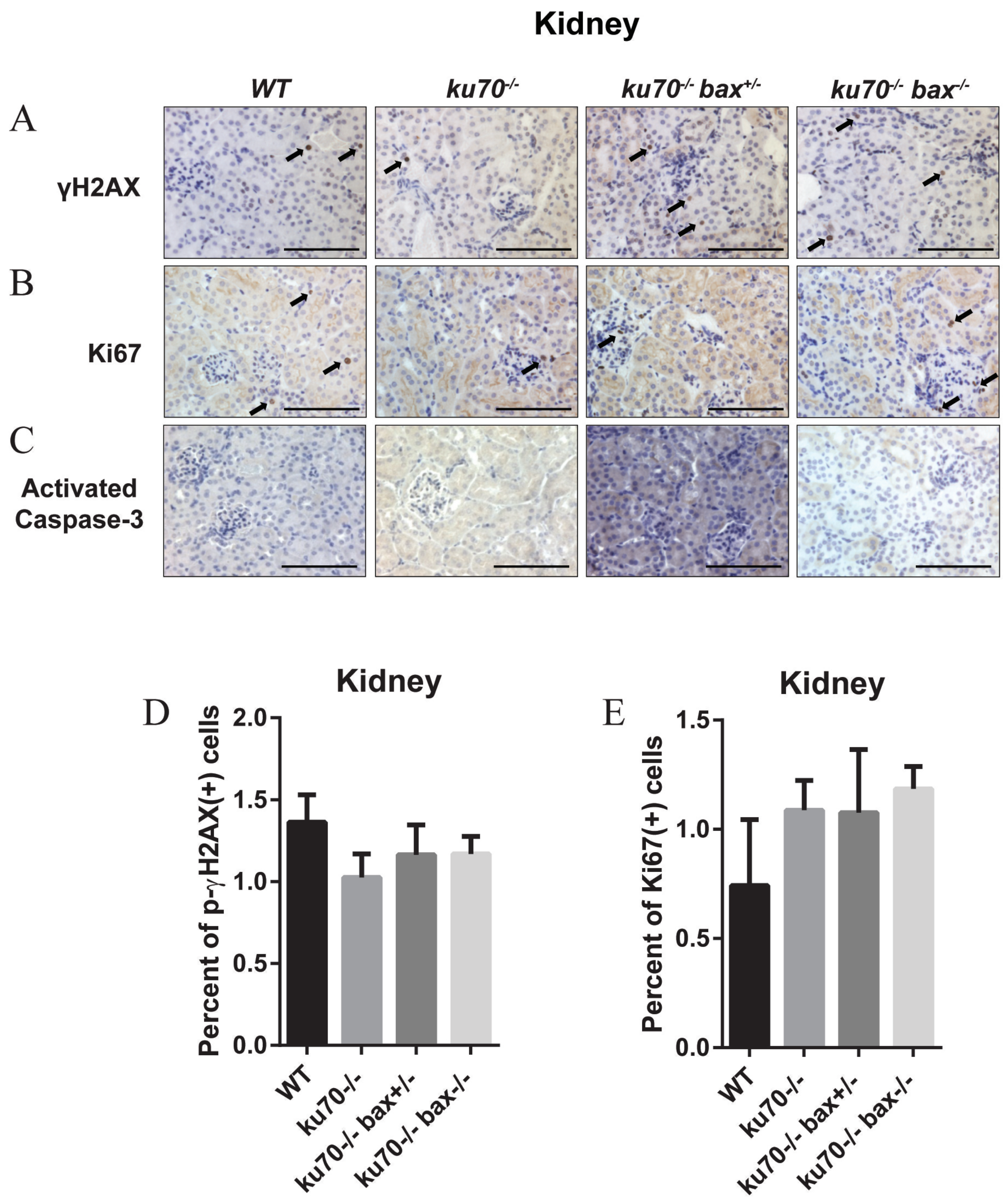

Figure S9. The absence of Ku70 did not lead to increased DNA DSBs in the kidneys. Staining of (A) phospho-γ-H2AX, (B) Ki67, and (C) activated caspase-3 were similar in all groups. Mice at similar ages (3-4 months old) were analyzed. The scale bar represents 250 μm. Quantification of (D) phospho-γ-H2AX and (E) Ki67 were not significantly different among all groups.
