# Supplementary material for: Bax deficiency extends the survival of Ku70 knockout mice that develop lung and heart diseases
Source: Cell Death Dis. 2015 Mar 26;6(3):e1706–. doi: 10.1038/cddis.2015.11 (PMC4385910; doi:10.1038/cddis.2015.11)
Supplement: Supplementary Figure S10 [file cddis201511x12.pdf]

Figure S10

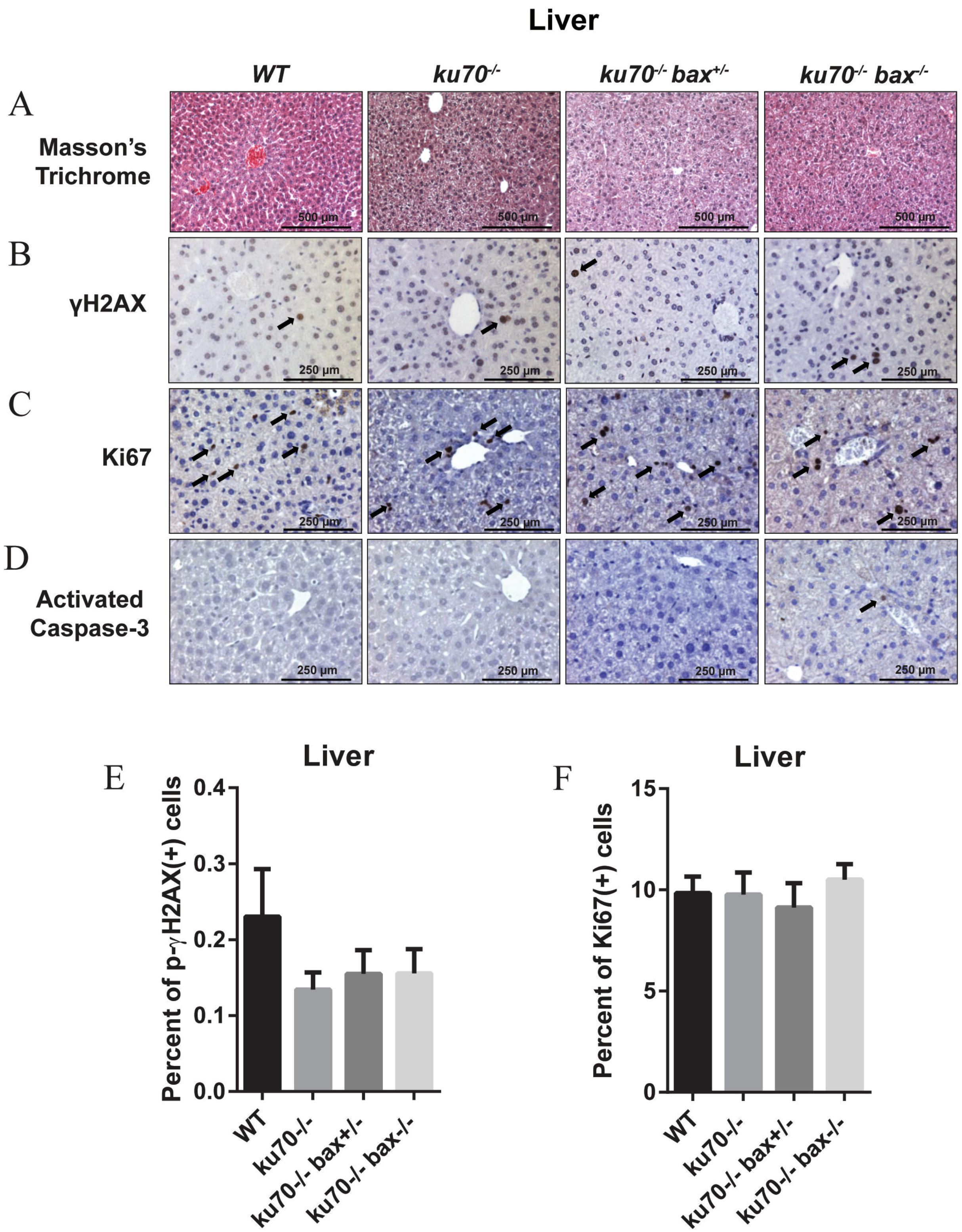

Figure S10. The absence of Ku70 did not lead to fibrosis or increased DNA DSBs in the liver. Staining of (A) Masson's trichrome (B) phospho-γ-H2AX, (C) Ki67, and (D) activated caspase-3 were similar in all groups of 3-4 month old mice. Quantification of (E) phospho-γ-H2AX and (F) Ki67 were not significantly different among all groups.
